# State-dependent representation of stimulus-evoked activity in high-density recordings of neural cultures

**DOI:** 10.1038/s41598-018-23853-x

**Published:** 2018-04-03

**Authors:** Thierry Nieus, Valeria D’Andrea, Hayder Amin, Stefano Di Marco, Houman Safaai, Alessandro Maccione, Luca Berdondini, Stefano Panzeri

**Affiliations:** 1 NetS3 Laboratory, Neuroscience and Brain Technologies Department, Istituto Italiano di Tecnologia, Genova, Italy; 2 Neural Computation Laboratory, Center for Neuroscience and Cognitive Systems @UniTn, Istituto Italiano di Tecnologia, Rovereto, Italy; 3 Department of Neurobiology, Harvard Medical School, 02115 Boston, Massachusetts USA; 4 Department of Biomedical and Clinical Sciences “Luigi Sacco”, Università di Milano, Milano, Italy; 5 Present Address: Scienze cliniche applicate e biotecnologiche, Università dell’Aquila, L’Aquila, Italy

## Abstract

Neuronal responses to external stimuli vary from trial to trial partly because they depend on continuous spontaneous variations of the state of neural circuits, reflected in variations of ongoing activity prior to stimulus presentation. Understanding how post-stimulus responses relate to the pre-stimulus spontaneous activity is thus important to understand how state dependence affects information processing and neural coding, and how state variations can be discounted to better decode single-trial neural responses. Here we exploited high-resolution CMOS electrode arrays to record simultaneously from thousands of electrodes in *in-vitro* cultures stimulated at specific sites. We used information-theoretic analyses to study how ongoing activity affects the information that neuronal responses carry about the location of the stimuli. We found that responses exhibited state dependence on the time between the last spontaneous burst and the stimulus presentation and that the dependence could be described with a linear model. Importantly, we found that a small number of selected neurons carry most of the stimulus information and contribute to the state-dependent information gain. This suggests that a major value of large-scale recording is that it individuates the small subset of neurons that carry most information and that benefit the most from knowledge of its state dependence.

## Introduction

Processing of external stimuli in neural circuits does not depend only on the stimulus presented as input to the circuit, but also on a number of internal neural and network variables often denoted as the “state” of the circuit^1,2^. Internal state variables that may influence the neural responses to an external stimulus include the neural activity still reverberating from the presentation of previous stimuli^1,3^, changes in the activity of the neuromodulatory nuclei that regulate behavioural states such as attention or arousal^4^, and intrinsic ongoing fluctuations of the excitability of a local or a large-scale network^5,6^. Changes of these state variables are reflected into changes of the spontaneous activity of the circuit prior to the application of the external stimulus^4,6–13^. Understanding how neural responses to stimuli depend on the network state variables captured by the ongoing activity is important for several reasons. It may help revealing the internal network context within which neurons operate^10^, thus helping in unravelling the mechanisms for complex, context dependent neural computations. It may give us clues on how the brain combines information from new sensory cues with information already present in neural activity. It can also shed light on the constraints under which neural population codes operate. A strong state-dependence of the response may either imply that populations have to transmit information only using codes that are robust to state fluctuations, or that, alternatively, downstream areas need to extract variables indicating the current network state and use state-dependent decoding to interpret population activity^14^. Finally, it can help to understand how sources of trial-to-trial variations, or “noise” that is shared among neurons recorded by different electrodes, can be modelled and then discounted in order to improve the performance of algorithms to extract information from neural activity^4^. This is important both to place bounds on the information that can be decoded from neural activity and to improve the performance of brain-machine interfaces.

State-dependent information processing has been studied at the level of small neural populations. However, it may involve a wide range of spatiotemporal scales of neural activity that are difficult to simultaneously access experimentally *in-vivo*. Reduced *in-vitro* models that exploit recent developments of large-scale recording multi-electrode arrays^15^ might therefore play a valuable role to study state-dependent coding at the scale of entire networks from which thousands of neurons are sampled. In this study, we take advantage of these large-scale *in-vitro* recordings to investigate the state dependent processing of stimulus information in cultured hippocampal neural networks. We delivered low-frequency trains of electrical stimuli (at 0.2 Hz) to the network from multiple, randomly selected on-chip electrode sites. Both ongoing and electrically evoked spiking network activities were acquired from 4096 closely spaced microelectrodes of CMOS-MEAs^16^. These devices allow almost complete sampling of the neuronal spiking activity in these networks^17^.

In this study, we first investigated how different features of the network population responses encode stimulus information. Second, to assess state dependence of neural information processing, we investigated whether knowledge of state variables, defined from measures of ongoing pre-stimulus spiking activity, increased the information that could be extracted from neural responses. Third, to mathematically describe the state dependence of neural responses with simple models, we investigated whether network responses can be predicted by a model consisting of a linear combination of the spontaneous ongoing pre-stimulus activity and of the stimulus-evoked activity. Fourth, we tested our results in a modulated firing regime by manipulating the cell cultures with norepinephrine, a neurotransmitter that was shown to decrease the network synchrony both *in-vivo*^18,19^ and *in-vitro*^20^. Finally, by leveraging our access to thousands of simultaneously recording electrodes, we investigated whether the information about a stimulus set can efficiently be decoded when considering the responses of a relatively small group of neurons^21^.

## Results

### Large-scale neuronal recordings of spontaneous and electrically evoked activities in cultured networks

**Figure 1:**
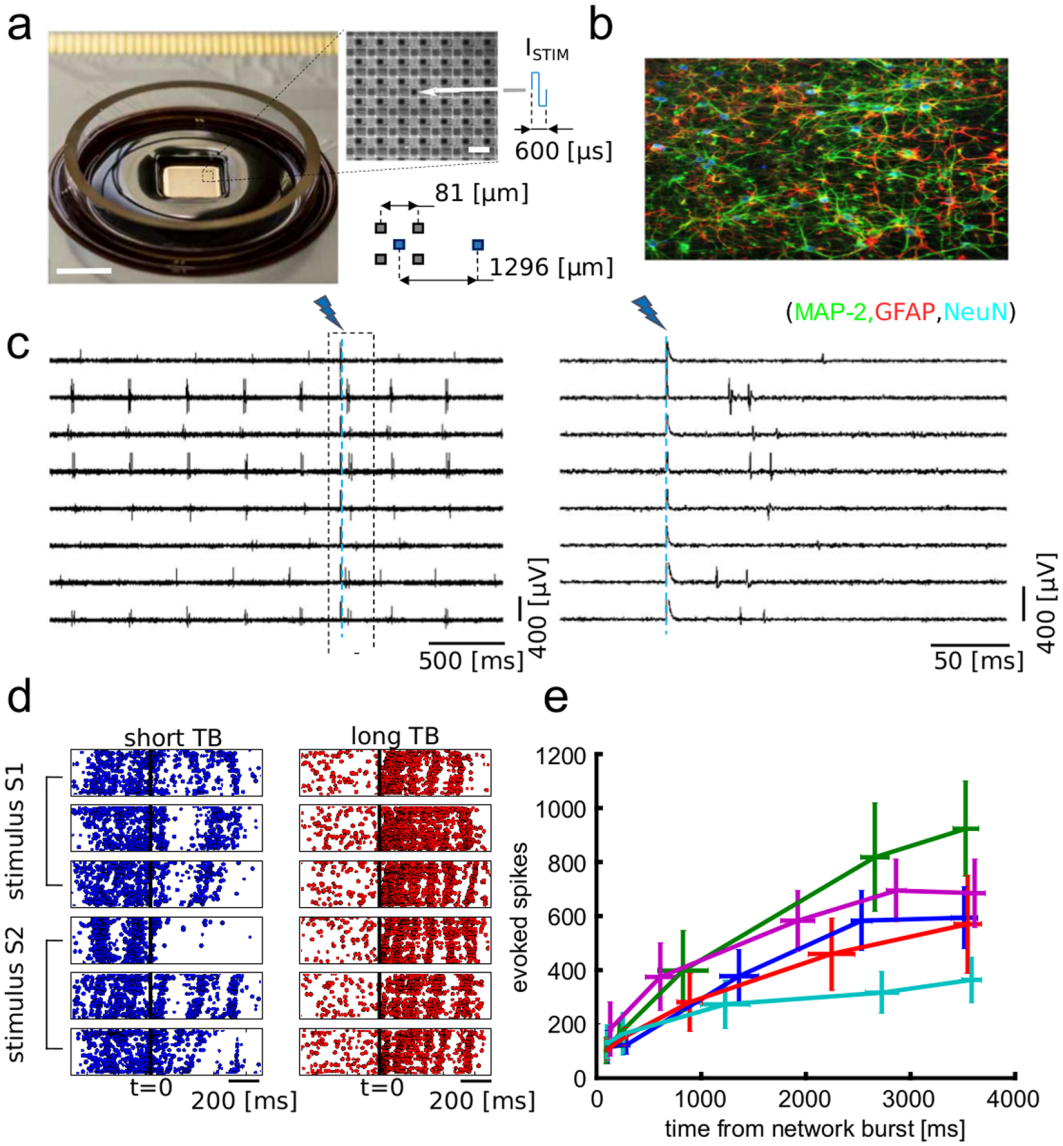
High-resolution recordings of ongoing activity and electrically evoked responses in neuronal networks. (**a**) View of a CMOS-MEA chip and close up on the electrode array. The arrow indicates an electrode for electrical stimulation. Scale bars represent 5 mm (left) and 80 µm (close-up, right). (**b**) Immunofluorescence image of a hippocampal neuronal network, obtained in separate pilot experiments in which neurophysiology was not performed, grown on-chip for 18 DIVs and stained for MAP-2 (green), a neuron-specific marker for dendrites, GFAP (red), a marker for astrocytes and NeuN (blue), a maker for neuronal nuclei. (**c**) Raw traces (left) and close-up around the stimulation instant (right) of representative electrodes showing ongoing and evoked spiking activity. (**d**) Raster plots illustrating the difference between evoked network response obtained with stimuli delivered at short (left) and long (right) time TB. Plots for two stimuli (S1, S2), delivered from two different electrodes, and for three stimulation trials are reported. As shown, for short TB (left) the response to a stimulus delivered at t = 0 is weaker than the response obtained for stimuli delivered at long TB (right). (**e**) Quantification of the averaged evoked number of spikes over a time window of 100 ms after stimulation as function of TB (8 different stimuli, 60 trials per stimuli). Each trace refers to a different cell culture. The TBs were grouped in quintiles. The reported mean and SEM on the x axis are computed over the quintiles of the TB distribution. The mean and SEM on y axis are computed on the corresponding evoked spikes.

We recorded ongoing and electrically-evoked neuronal spiking activity in n = 5 primary hippocampal neuronal cultures grown for 24 days *in-vitro* using high-density CMOS multi-electrode array (CMOS-MEAs) chips (Fig. 1a and b). These devices provide 4096 simultaneously recording electrodes (81 μm pitch) and 16 individually addressable electrodes (1296 μm pitch) for delivering electrical stimuli. The spontaneous network activity and evoked spiking responses were recorded from each electrode at 7.7 KHz/electrode (Fig. 1c). Biphasic current stimuli (600 μs in duration, amplitude tuned between 200–600 μA) were delivered at a frequency of 0.2 Hz from 8 spatially distributed sites using randomized sequences of the stimulation sites (total of 60 trials for each stimulation electrodes). As we have previously shown in these devices^22^, the artefacts of the electrical stimuli were localized in areas of ~100 μm in diameter around the stimulation sites. Therefore, by providing artefact-free recordings from electrodes close to the stimulation sites we could spatially and temporally resolve electrically evoked spiking responses at short lags (<3 ms) from the stimulation time. As previously reported^23^, the high spatiotemporal resolution of our recordings allowed us to show that spontaneous network activity was characterized by propagating waves of spikes (or network bursts) interleaved by inter-burst periods of sparse spiking activity (see the pre-stimulation activity in the raster plots of Fig. 1d). Electrical stimuli evoked propagating network-wide bursts of spiking activity that visually resembled the bursts observed during spontaneous activity (Fig. 1d). Different trials of electrical stimulation from different sites, however, elicited different network responses. Visual inspection of the network activity suggested to us that the trial-to-trial variability of the responses to the same electrical stimulus was modulated by the time (TB) of the stimulus application from the last spontaneous network burst observed prior to stimulation. For shorter TBs (TB < ~500 ms), the evoked responses were weaker and less spatially precise (Fig. 1d left). For longer TBs (TB > ~500 ms), the evoked responses were stronger and the stimulation induced multiple sequential waves of spikes propagating in the network (Fig. 1d right). To evaluate how the electrically evoked spiking activity depended on TB, we computed the trial-averaged number of spikes elicited over a time window of 100 ms for stimuli delivered at different ranges of TB. Results, averaged over all stimuli but plotted separately for individual cultures, show (Fig. 1e) that responses to stimuli delivered at long TB were consistently stronger than responses to stimuli delivered at short TB. In sum, both qualitative and quantitative analyses suggest that the variable TB could represent a suitable parameter to describe the state of the network. In the next sections, we will further inspect quantitatively the role of TB and other putative state variables in modulating the network responses to the stimuli.

### The time (TB) between stimulation and last spontaneous network burst is the most informative network state variable

Initial inspection of the data, as reported above, suggested that the time TB between stimulation and the last spontaneous network burst is a variable that strongly influences post-stimulation responses. In this section, we corroborate this intuition by selecting a number of other possible candidate state parameters θ, besides the already mentioned TB, and by comparing quantitatively the influence of different pre-stimulus ongoing activity parameters on stimulus-specific post-stimulus responses. As possible network state parameters, we considered: the number of spikes in the last network burst preceding the stimulation (NSP), the ignition site (IS) of the last network burst, the network burst rate (NBR), the amplitude (ampl) of low-frequency (6–12 Hz) fluctuations of network activity, the phase (phase) of such low-frequency fluctuation, and the mean firing rate (MFR).

To quantify the effect of each such pre-stimulus state variable θ on the stimulus specificity of the responses, we used information theory^24–26^. We computed and compared two different kinds of information^4^. First, we computed the information *I*(*S*; *R*),about which stimulus s (out of a set of *S*) was presented, that was carried by the post-stimulus response *r* in the same trial (see Eq. 3). Second, we quantified the information *I*(*S*; *R*, *Θ*), about which stimulus was presented, that was carried from the joint observation, in the same trial, of post-stimulus response *r* and pre-stimulus state parameter θ (see Eq. 4). Finally, to quantify the effect of the state variable on the stimulus-specificity of the neural response (and thus the stimulus information they carry), we used the difference *I*(*S*; *R*, Θ) − *I*(*S*; *R*), referred to as information gain. In our data, we verified that, as expected by the fact that θ is defined as a pre-stimulus variable, θ does not carry per se any stimulus information (see Supplementary Fig. S1). Under this condition, θ can carry stimulus information only through synergistic interaction with the post-stimulus response *r* (see Methods) and the quantity *I*(*S*; *R*, Θ) − *I*(*S*; *R*) is large when the state variable θ modified the stimulus-specificity of the neural responses, while it is small when θ did not modulate the stimulus specificity of the neural responses (see Methods). Importantly, this measure has the advantage of concentrating on the stimulus-specific effect of θ on r. Thus, the measure *I*(*S*; *R*, Θ) − *I*(*S*; *R*) is expected to be zero if Θ had a non-stimulus specific effect (or no effect at all) on the response probabilities and to be positive otherwise. To express this information gain in proportional terms, we also introduced the percentage information gain due to state dependence, defined as the ratio between *I*(*S*; *R*, Θ) − *I*(*S*; *R*) and *I*(*S*; *R*), multiplied by 100.

**Figure 2:**
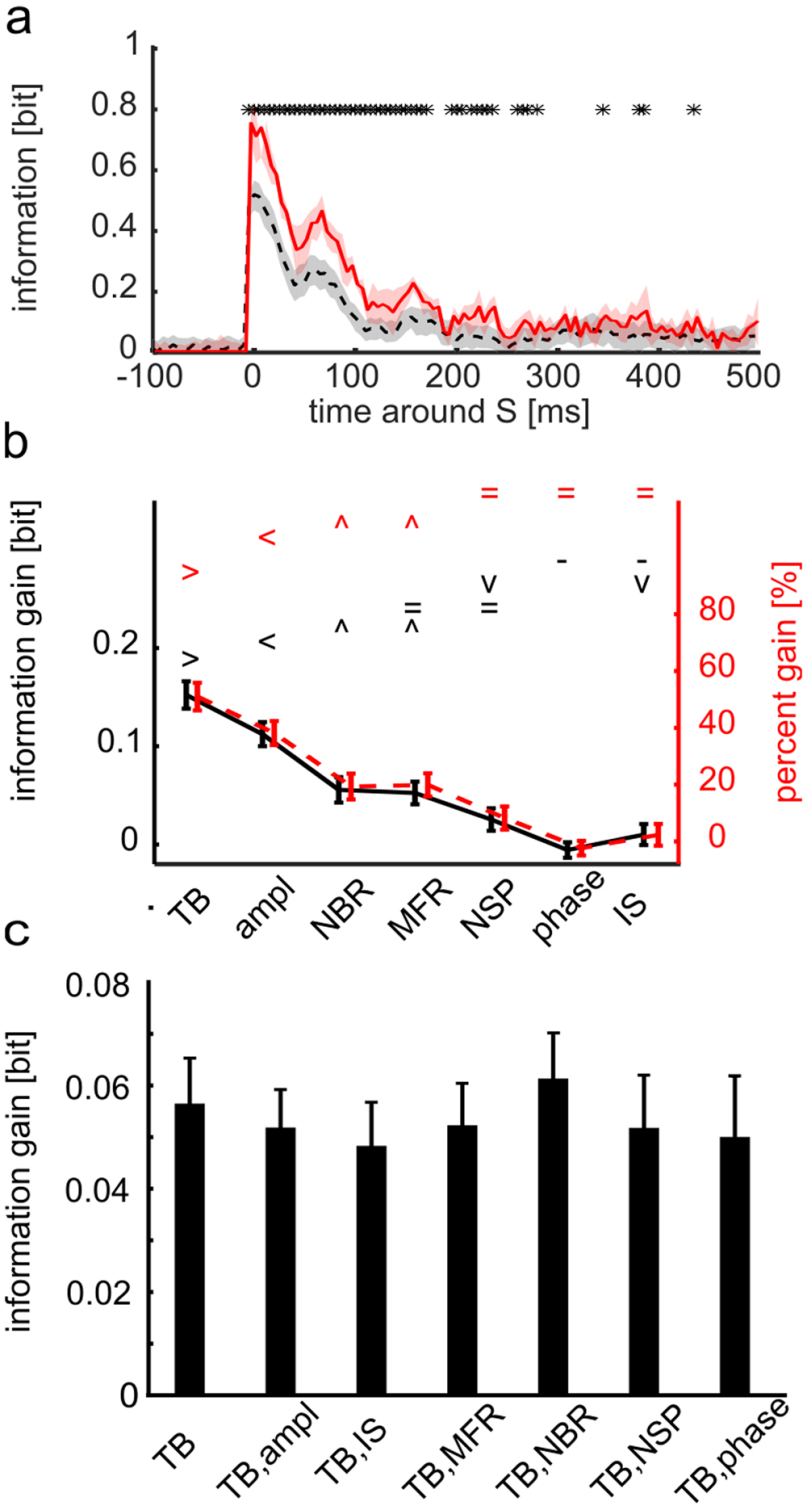
Post-stimulus response carries higher information about the stimulus when considered together with pre-stimulus state variable θ. (**a**) Time course of the across-sessions average (n = 5) of the information *I*(*S*; *R*, Θ) about stimuli carried by responses *R* and state variables Θ = *TB* (red solid line) and the across-sessions average of the information *I*(*S*; *R*)computed when destroying the information in the variable Θ by shuffling its values across trials (black dashed line). The red area delimits SEM across sessions of *I*(*S*; *R*, Θ). The grey area around *I*(*S*; *R*) delimits the area between the 5th and 95th percentiles of the average across sessions of the distribution *I*(*S*; *R*, Θ_*SH*_) obtained with N = 200 random permutations of state variables Θ across trials. Stars indicate time points in which *I*(*S*; *R*, Θ) is significantly higher than *I*(*S*; *R*) (one-tailed permutation test, p < 0.05 FDR corrected). (**b**) Mean and SEM across experiments of information gain (black line) and percentage information gain (red dashed line) in a [0 100] ms time window after the stimulus, for different state variables. Symbols { > < ^ = −} mark data groups that have similar means (Tukey’s HSD, p < 0.05), see Supplementary Information. Black and red symbols indicate not significantly different means for, respectively, information gain (F(6, 639) = 76.06, p = 10^−73^ one-way between subject ANOVA followed by Tukey’s HSD multiple comparison test) and percentage information gain (F(6, 639) = 46.84, p = 10^−48^, same test). State variable TB has information gain and percentage information gain significantly higher than other considered state variables. (**c**) Mean and SEM across experiments of the decoded information gain summed in a [0 100] ms time window after the stimulus, for the TB state variable and corresponding bi-dimensional state variables (where TB is paired with either ampl, IS, MFR, NBR, NSP and phase). There are no significant different sessions averages of decoded information for any comparison between bi-dimensional and one-dimensional state variables (F(6, 28) = 0.18 with p = 0.98, one-way between subject ANOVA).

We investigated how this information changed when considering all the above described candidates for the pre-stimulus state variables Θ. For this analysis, and throughout this subsection, we focused on the simplest and most traditional representation of neural responses, *r*, that is the total spike rate (or multi-unit-activity, shortened as MUA hereafter) of the network activity computed as function of the post-stimulus time. The average across sessions of the information *I*(*S*; *R*) that MUA carried about stimuli, peaks at around ~20 ms and dropped almost to zero after ~500 ms from the stimulation time (Fig. 2a, dashed black curve). Averaging across sessions in the [0 100] ms post-stimulus interval, the information *I*(*S*; *R*) had a value of 0.29 ± 0.05 bits. The temporal profile of information was highly reproducible across experiments (Supplementary Fig. S2). We then evaluated the effect of using as state variable *θ* = *TB*, the time between the last spontaneous burst and stimulation, on the stimulus information carried by post-stimulus MUA. The increase in information due to the knowledge of the state variable *θ* = *TB* was larger in the earlier parts of the neural response, particularly in the [0 100] ms range, where also the information *I*(*S*; *R*) was larger (Fig. 2a and Supplementary Fig. S2). Averaging across sessions in the [0 100] ms post-stimulus interval, the information *I*(*S*; *R*, Θ) had a value of 0.44 ± 0.06 bits. The information gain due to knowledge of state dependence was significant (p < 0.05; permutation test; FDR corrected) at 78% of all time points in the first 300 ms post-stimulus (Fig. 2a).

To understand which of the possible candidate state variables Θ (out of the set we considered) had a larger influence on the stimulus dependence of neural responses, we computed the information gain due to the knowledge of the state variable *θ* that could be obtained above and beyond the stimulus information that could be obtained by *r*. Results (Fig. 2b) show that TB was the variable that gave the highest information gain, both in absolute and in percentage terms. The information gain with TB, averaged across sessions in the [0 100] post-stimulus window, was 0.15 ± 0.01 bits, and was 51% in percentage terms. Other putative state variables, such as the instantaneous amplitude and phase of low-frequency MUA oscillations, led to lower information gains (Fig. 2b).

We then considered whether the information gain provided by other candidate state variables was complementary to that provided by TB, or instead whether TB was sufficient to account for the whole information gain. To address this question, we computed the gain of information by considering bi-dimensional state variables made of TB and any one of the other candidate state variables listed above. We compared this gain of information with the information gain obtained when considering as state variable only TB. This calculation was not possible, due to data sampling issues, with the direct calculation of information from the response probabilities, Eqs (3 and 4). We thus performed it by first using a decoding procedure to reduce the dimensionality, and then computing the information in the confusion matrix of this decoder^25^ (see Supplementary Methods, Section “Computation of information from the confusion matrix of a decoder”). This calculation quantifies how well we decoded which stimulus was being presented based on the considered quantification on neural activity. The computation of information from the decoding matrix (Supplementary Eq. S1) has the advantage of being more data robust^25^ than the direct calculation of total information in neural activity from Eq. 1, used above. This robustness allowed us to consider more state variables in this analysis. However, computing information through a decoder captures only a part of the total information in neural activity measured directly with Eqs 3 and 4 (see Supplementary Information, Section “Computation of information from the confusion matrix of a decoder”). Indeed, in our data we found that both the total stimulus information decoded through knowledge of post-stimulus MUA response and TB, the information gain obtained with TB when computed through the confusion matrix had a lower value (0.23 ± 0.01 and 0.06 ± 0.01 bits, respectively) than that obtained when computed directly from the response probabilities (Eqs 3 and 4) and reported in Fig. 2a,b. However, and importantly, this decoding analysis showed that the information gain obtained when decoding jointly more than one state variable was not higher (p = 0.98, one-way between-subjects ANOVA) than the one obtained when considering TB alone (Fig. 2c). This means that all other state variables give an information gain that is redundant to that obtained with TB. An intuitive explanation for this finding is that the other state variables that showed some information gain, such as NBR and MFR, were also strongly correlated with TB (NBR Pearson correlation = −0.81 ± 0.01, MFR Pearson correlation = −0.40 ± 0.12), and the other ones were not providing any appreciable information gain (Fig. 2b). Therefore, hereafter we will consider only *θ* = *TB* as state variable.

### Post-stimulus network activity variables carrying the most stimulus information and gaining the most from considering state dependence

**Figure 3:**
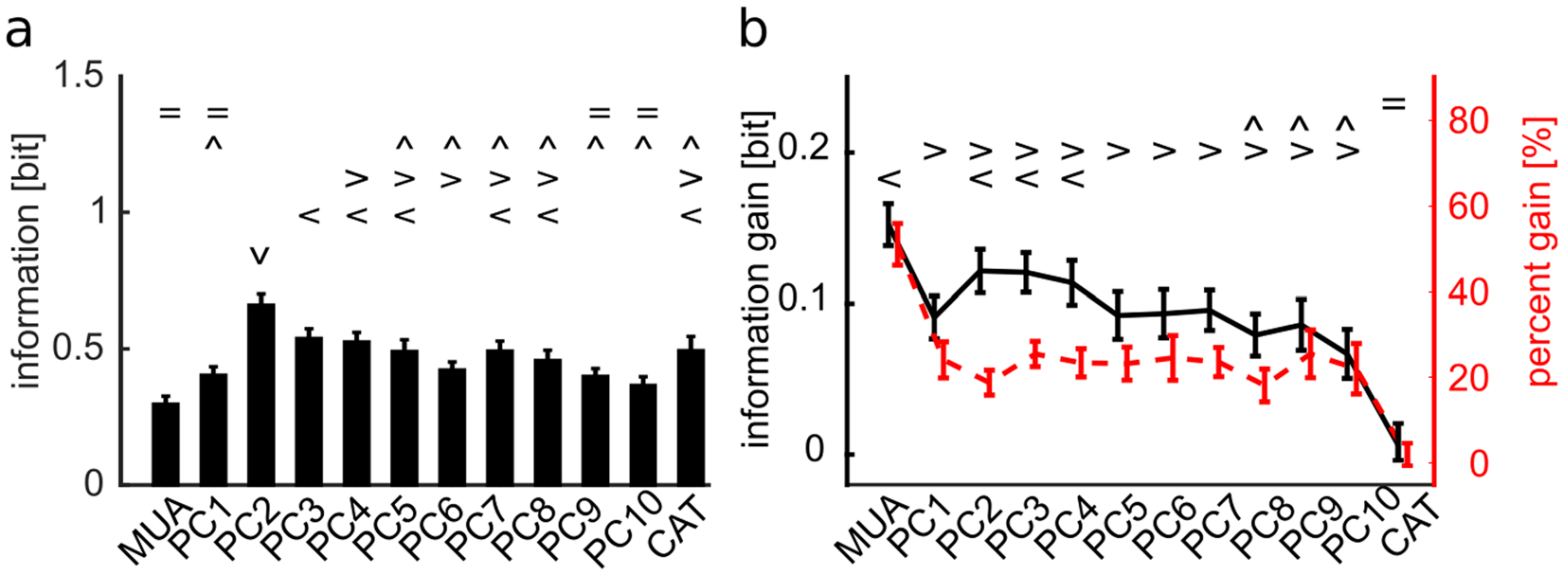
Stimuli information carried by R changes according to different post-stimulus network responses. (**a**) Mean and SEM across experiments of *I*(*S*; *R*) summed over 100 ms post-stimulus time interval for different network responses *R*. Symbols { = ^ > < v} mark data groups that have similar means (Tukey’s HSD, p < 0.05), see Supplementary Information, Different symbols indicate not significantly different means for information *I*(*S*; *R*)(F(12, 1287) = 30.45, p = 10^−62^, one-way between subject ANOVA followed by Tukey’s HSD multiple comparison test). PC2 response carries the highest stimulus information. (**b**) Mean and SEM across experiments of information gain (black line) and percentage information gain (red dashed line) for different network responses. Symbols { < > ^ = } mark data groups that have similar means (Tukey’s HSD, p < 0.05), see Supplementary Information. Black symbols indicate not significantly different means for information gain (F(12, 1287) = 14.99, p = 10^−30^, one-way between subject ANOVA followed by Tukey’s HSD multiple comparison test). Differences between response measures are not significant for the percentage information gain (F(12, 1287) = 1.5, p = 0.11).

In the above analysis, we concentrated on the information carried by the total multi-unit-activity of the network. However, little is yet known about neural networks read out the output of other networks. In particular, it may be possible that a neural readout may weigh spikes of neurons at different locations with a different weight. To study in more depth the effect of the network state variables Θ = *TB* on different aspects of the neural population responses to different stimuli, and to check if state dependent gain was stable across different quantifications of the neural response, we computed the information gain and the percentage information gain for different representations of the population response feature that may carry stimulus information. The features of the neural population responses that we considered were the centre activity trajectory (CAT) in each time bin, which takes into account one prominent aspect of the spatial distribution of the propagating neural activity, and the projections of the neural activity in each time bin along one of the 10 first spatial Principal Components (PC) of the neural activity, a variable that takes into account the spatial structure of the population activity. Each of these different features of the neural population activity captures a different and potentially interesting aspect of its spatial structure. In particular, PCs provide a simple way to explore different ways to weigh neural activity that account for different portions of the variance of neural population responses. Because of this, PCs have been a popular tool to study candidate neural codes for decades^27^. All measures, described in Methods, were based on the network spiking activity discretized in 20 ms bins because this time scale were found empirically by us to be short enough to capture the major time scales of the observed variations and long enough to ensure robust calculations of the considered quantities (see also^28,29^). We found (Fig. 3a) that several PCs had much higher stimulus information *I*(*S*; *R*) than the one computed with the MUA. In particular, while PC1 had positive weights (Supplementary Fig. S4) and was approximately as informative as MUA and highly correlated with it (Pearson correlation = 0.91 ± 0.01), PC2 (a more spatially structured component than PC1, see Supplementary Fig. S4 for examples of spatial structures) was the one that, across sessions, had the highest information. These results are consistent with earlier findings that the responses of cultured networks to electrical stimulations are highly spatially structured^29^. On average over sessions, the information in higher PCs was larger than MUA up to the 8^th^ PC. The CAT representation carried an amount of information about the stimuli similar to that carried by PC1. The relatively high information carried by CAT reflected the fact that the position of the centre of mass of the evoked activity depended on the location at which the stimulus was applied^29^. We next investigated whether the information gain due to the modulation of the stimulus-response relationship by the state variable TB was different for the different spatial representations of neural responses. Results (Fig. 3b) showed that this information gain, expressing the strength of state modulations, was the highest for MUA but was also considerable for all PCs. One important exception was that the CAT showed a negligible state modulation. We note that we verified that the results on the information gain computed with state variables Θ others than TB and response variables *R* other than MUA were robust, i.e. the state variable TB remained the variable giving the most information gain for all considered choices of features quantifying population responses.

### Linear models of the dependence of network responses to stimuli on the pre-stimulus state variable TB

Results in previous section showed, with information theoretic measures, that the state variable TB strongly modulated the stimulus-response relationship of the network. However, these previous results did not describe how the state variables actually modulated the responses to different stimuli and how they affected the responses of the network in each single trial. Here, following previous works^6,30,31^, we used linear models to describe the stimulus-response relationship and its modulation by the state variable θ = *TB* on single trial response *r*. To characterize the modulation of TB on *r* we used MUA as the feature of *r* quantifying single trial neural responses, and we investigated whether the trial-to-trial variations around the stimulus-specific mean of each single-trial response could be described as a linear function of the state variable TB. Specifically, we considered the variation *dr* of the response *r* in each trial around the trial-averaged response to the stimulus presented in that trial:

1 $$dr(TB)=\langle r(TB,s)-{\langle r(TB,s)\rangle }_{s}{\rangle }_{s}$$

**Figure 4:**
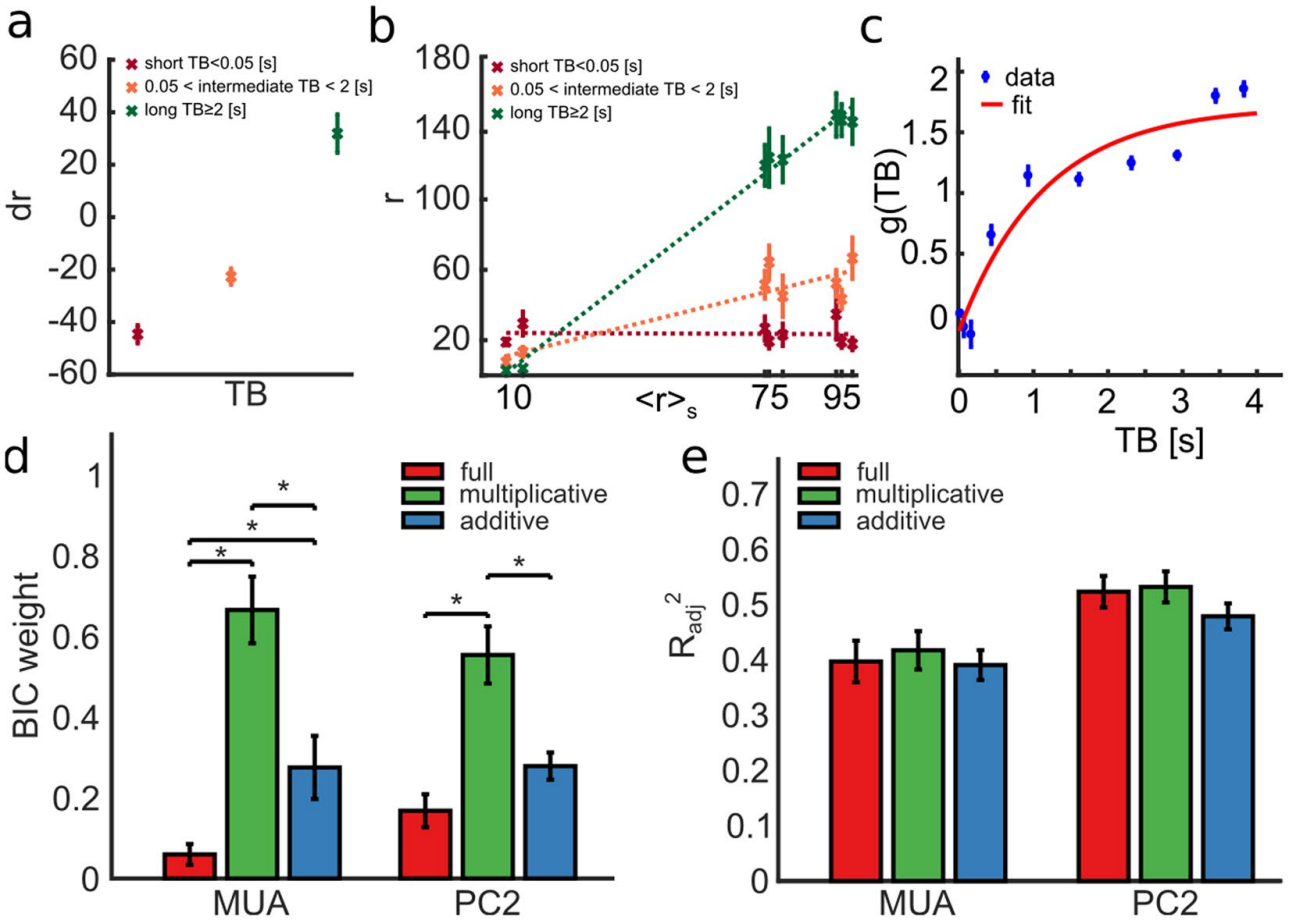
Single trial response r modeled as a linear combination of a stimulus-driven term and a noise term, both depending on state variable θ = TB. Panels A to C refer to the example recording session 208. All measures are computed in [0 100] ms time interval after stimulus. **(a)** Mean ± SEM across trials of trial-to-trial variability *dr* of MUA as a function of TB. State variable values are divided in three intervals: low TB if TB < 0.05*s*, high TB if TB ≥ 2s, intermediate TB otherwise. *dr* depends linearly on TB (Pearson correlation = 0.97, p = 10^−37^). **(b)** Single trial response *r* depends linearly on the mean response at fixed stimulus 〈*r*〉_*S*_ with a slope *g* that depends on TB. Along the x-axis, MUA responses 〈*r*〉_*S*_ are ranked according to the corresponding stimulus. On the y-axis mean response *r* across trials and SEM are reported for long, intermediate and short TB (lines represent best linear fits). **(c)** MUA gain parameter *g* of the purely multiplicative model as a function of TB; data points show mean and SEM of *g*(*TB*) for TB binned in 10 equi-populated intervals; red line shows bi-exponential fit to the data points. **(d)** Mean ± SEM across all experiments of BIC weights for, respectively, full model (red bars), purely multiplicative model (green bars) and purely additive model (blue bars). The BIC weight of the purely multiplicative model is significantly higher (1-way ANOVA followed by Tukey’s HSD multiple comparison test) than BIC weights estimated in other models when considering both MUA (F(2, 297) = 83.45, p = 10^−29^) and PC2 (F(2, 297) = 23.82, p = 10^−10^) as response features. **(e)** Mean and SEM across all experiments of the coefficients of determination R^2^_adj_ between responses *R* and *R*_*model*_ adjusted for the number of parameters used in the model is reported for MUA and PC2 response representations. For most of the response representations, the coefficient of determination is higher for the purely multiplicative model than for the full or purely additive model, although the means ${R}_{adj}^{2}$ of different models are not significantly different (1-way ANOVA, p = 0.2) for the evaluated response representations.

We computed this quantity for three ranges of TB values (short, intermediate, and long), considering as neural response feature the [0 100] ms post-stimulus window where the state modulation is larger (see Figs 3b and 2a). Figure 4a–c show results from an example experiment (session 208). When pooling all trials to all stimuli, we found that TB modulated (1-way ANOVA, F(2, 477)=100.4, p = 10^−37^) the response MUA in a single trial by increasing (respectively decreasing) the response, with respect to the stimulus-specific trial-averaged response, when TB was long (respectively short) in that trial (Fig. 4a). This is compatible with both an additive and a multiplicative effect of TB. To shed light on these two alternatives, we also investigated how the state parameter TB affected the trial-averaged MUA response to each stimulus. We computed trial-averaged responses to each stimulus and ranked them in each experiment according to their average value. We then separately computed stimulus-specific averages only to trials with short, intermediate or long values of TB, and fitted the trial-averaged firing rate dependence on each stimulus with a linear curve separately for each TB class. As shown in Fig. 4b for the example recording session, we found that a longer (respectively shorter) TB corresponded to a higher (respectively lower) stimulus response slope, thus suggesting that the time between the last burst and the stimulus application modulated the gain of the stimulus-response relationships.

Based on the above results, we described the effect of the state variable on *r* with a simple additive-multiplicative model of the type used to describe state dependence in cortical networks^6,30^, as follows:

2 $${r}_{model}=g(TB){\langle r\rangle }_{S}+b(TB)$$

The linear model in Eq. (2) describes a single-trial neural response *r*_*model*_ as a stimulus-driven term *r*_*s*_ (that is the mean response at fixed stimulus) with a multiplicative term *g* and an additive term *b* that both may depend on the state variable TB. In this model, the state variable determines the response in each trial by adding state-dependent noise and/or by rescaling the stimulus-response relationship. For each trial and for each experiment, and separately for each post-stimulus time, we estimated the additive *b*(*TB*) and multiplicative *g*(*TB*) model parameters by best fit to the data, with a four-fold cross-validation procedure (see Methods). Moreover, we considered for this specific analysis two different features for defining the neural response *r*: the MUA (the simplest and most widely used definition of response, which also has the largest state dependent information gain) and PC2 (the one that had the largest information).

To understand how necessary the additive and multiplicative components were, we fitted to the single trial data three variants of the model in Eq. (2): a “full model”, that included both multiplicative and additive terms as free parameter; a “purely multiplicative model” that contained only gain *g* as free parameter (with *b* set to zero); and a “purely additive model” that contained only baseline *b* as free parameter (with *g* set to one). The distribution of the best-fit gain parameters of the purely multiplicative model run using MUA as response variable *r* (and averaged across all trials and experiments in the [0 100] ms post-stimulus window) is shown in Fig. 4c, again for the example session, together with the fit to a bi-exponential function. For this estimation, we binned the state variable TB into 10 equi-populated intervals. The best-fit parameters of *g*(*TB*) showed a larger gain for longer TB values, compatible with the previous results showing that longer TB values enhanced the network responses to the stimuli. These results were robust across sessions (Supplementary Fig. S3). In particular, by applying a one-way ANOVA followed by Tukey’s HSD multiple comparison test, we found that the gain for long TB (TB >2 s) was, across all sessions, larger (p = 0.009) than the gain at intermediate TB (0.05 s < TB <2 s) and that the gain at intermediate TB is larger (p = 0.001) then gain at short TB (TB < 0.05 s). To evaluate the fitting performances of these different models we used the Bayesian information criterion (BIC). Higher BIC weights indicated better model performance. We found (Fig. 4d) that the BIC weight of the purely multiplicative model was significantly higher (1-way ANOVA, F(2, 297) = 83.45, p = 10^−29^ for MUA, and F(2, 297) = 23.82, p = 10^−10^ for PC2) than in the others models. For MUA, the BIC weights were 0.67 ± 0.08 for multiplicative; 0.27 ± 0.07 for additive and 0.06 ± 0.02 for the full model. For PC2, the BIC weights were 0.55 ± 0.07 for multiplicative; 0.28 ± 0.03 for additive and 0.17 ± 0.04 for the full model. As a consequence of the fact that the full model did not add explanatory power, we found that the model’s coefficient of determination adjusted for the number of coefficients ${R}_{adj}^{2}$ was as large for the multiplicative model as for the full model (Fig. 4e). These results indicate that the time interval between the last spontaneous burst and the stimulus affected the responses mainly with a multiplicative term, and that all increases of MUA and PC2 scores at longer TB could be explained by a gain rescaling rather than a background addition.

An advantage of an explicit model of state modulation, such as the linear models in Eqs (1,2), is that it can be used to predict each single trial response to a stimulus from the value of the pre-stimulus state variable in that trial. This prediction can then be subtracted from the single trial responses to reduce their variability at fixed stimulus and increase in this way the information they carry. To evaluate how effective this discounting was at increasing the stimulus information in neural responses, we subtracted the prediction of the linear model of the trial-to-trial response variability based on TB in that trial, and we computed the information from this response that discounts state induced variability. We considered initially both multiplicative, additive and full linear models.

**Figure 5:**
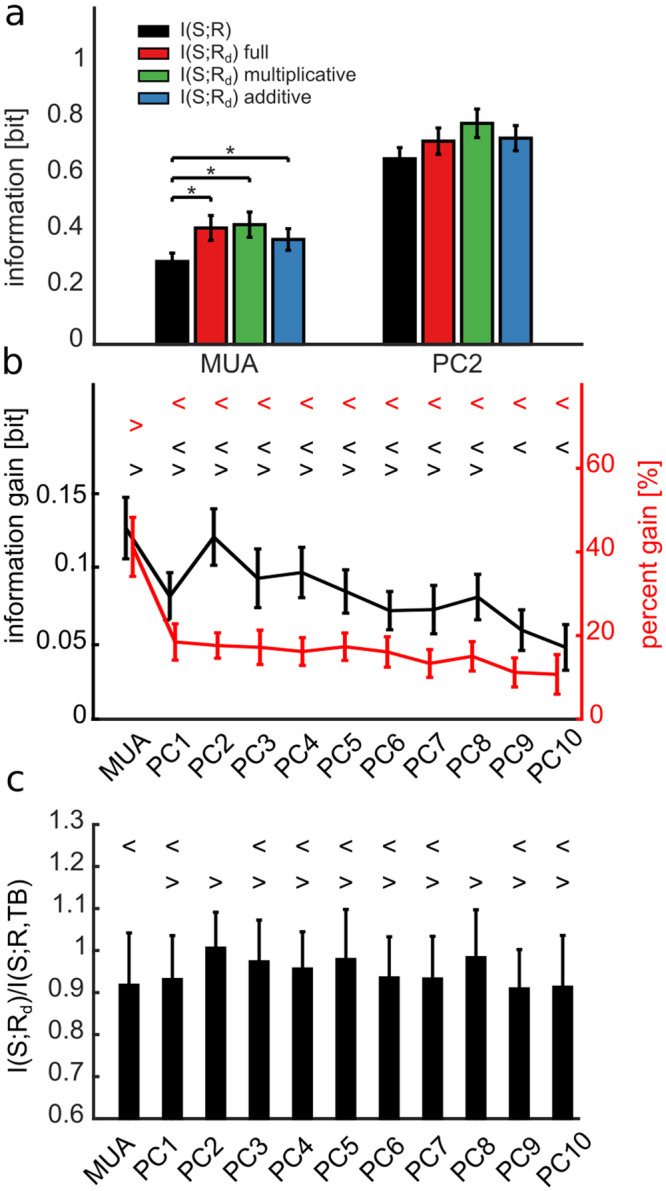
Discounting predicted trial-to-trial response variability from single trial response increases the stimulus information. (**a**) Mean ± SEM across all experiments of the mutual information between stimulus and response in a post-stimulus time window of [0 100] ms. We considered either MUA or PC2 as response representations. Information is computed before (*I*(*S*; *R*), black bars) and after (*I*(*S*; *R*_*d*_)) discounting from the single trial response *r* the trial-to variability predicted by, respectively, the full model (red bars), the purely multiplicative (green bars) and the purely additive (blue bars) model. Among the evaluated response features only MUA shows significant differences between *I*(*S*; *R*) and *I*(*S*; *R*_*d*_) for the full, multiplicative and additive models (black asterisk, F(3,396) = 8.58, p = 10^−5^, one-way between subject ANOVA followed by Tukey’s HSD multiple comparison test), whereas differences are not significant (p = 0.07) for PC2. (**b**) Information gain and percentage information gain for different response representations computed using the purely multiplicative model. Symbols {> <} mark data groups that have similar means (Tukey’s HSD, p < 0.05), see Supplementary Information. Black and red symbols indicate not significantly different means for, respectively, information gain (F(10, 1089) = 3.6, p = 10^−5^, one-way between subject ANOVA followed by Tukey’s HSD multiple comparison test) and percentage information gain (F(10,1089) = 7.9, p = 10^−12^). **(c)** Mean ± SEM across all experiments of the discounted information ratio, measured as the ratio between *I*(*S*; *R*_*d*_) and *I*(*S*;*R*, *TB*) (F(10, 1089) = 2.6, p = 0.003). Symbols {> <} mark data groups that have similar means (Tukey’s HSD, p < 0.05), see Supplementary Information.

Figure 5a shows the mean and SEM across all experiments of the mutual information between stimuli and responses computed after discounting state dependency in a 100 ms time window after stimulus when considering as response features MUA and PC2, the most used and the most informative response features. Consistent with our findings presented above (Fig. 4) that the purely multiplicative state dependence model (Eq. (2) with *b* = 0) was the one most effective at predicting neural responses, we found that no model performed better than the multiplicative one at discounting state dependence to gain information (Fig. 5a). When using this multiplicative model to discount state variability and testing it with MUA and all PCs as response features, we found that information increased significantly, both in absolute and in percentage terms, with respect to the information *I*(*S*; *R*) present before discounting, when both MUA and all PCs were used as response features (Fig. 5b). Being computed from knowing the value of both responses and state variables, the discounted information *I*(*S*; *R*_*d*_) is bounded from above by the information *I*(*S*; *R*, TB) by the data processing inequality. If the model captures all effects of state variables TB on the stimulus-specific responses, then the information *I*(*S*; *R*_*d*_) will be close to *I*(*S*; *R*, TB). Conversely, if the model captures only a small part of the relationships between stimulus, response and state variable, then the information *I*(*S*; *R*_*d*_) will be very small compared to *I*(*S*; *R*, TB). As shown in Fig. 5c, in our data we found that the ratio *I*(*S*; *R*_*d*_)/*I*(*S*; *R*, *TB*) was very close to one (0.91 ± 0.12 and 0.98 ± 0.07 when we took as response feature *R* and *PC2*, respectively). This suggests that our multiplicative linear model captures the vast majority of the stimulus-response-state relationships.

### Modulation of the state dependent processing by application of norepinephrine

We then wondered how state dependence changes when the spontaneous network firing regime changes. To investigate this, in addition to recording spontaneous and evoked activities from cell cultures in the basal condition, we pharmacologically manipulated the same neural cultures (n = 5) to alter their spontaneous firing.

**Figure 6:**
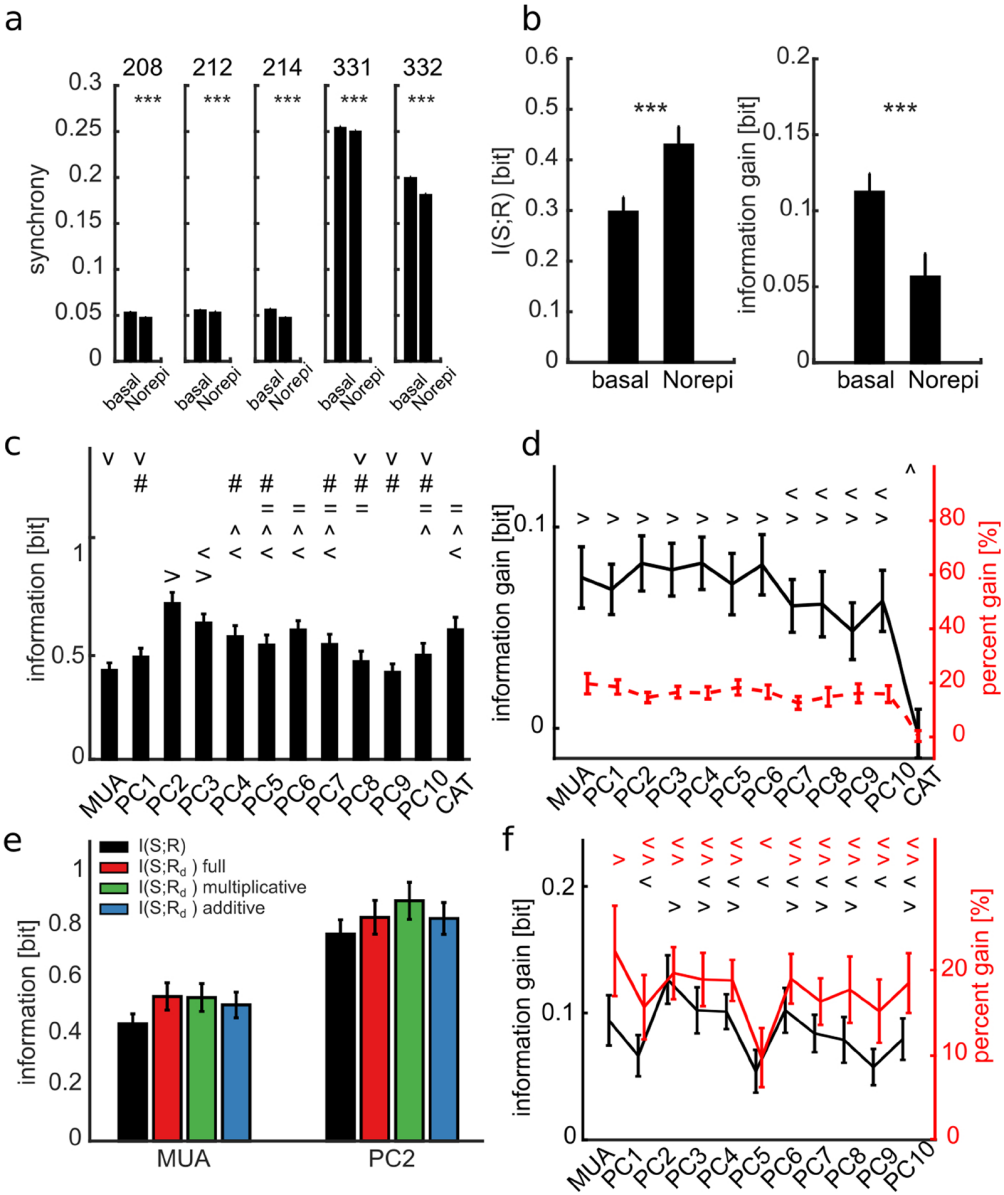
Cell cultures treated with norepinephrine display increased information and decreased information gain with respect to the basal untreated condition. (**a**) Each box-plot shows mean ± SEM of the spike synchronization coefficient computed over 100 randomly selected sets of 100 electrodes from the whole array. The synchronization coefficient decreases significantly when the norepinephrine drug is added in all tested cell cultures (two tailed t test, p = 10^−6^, p = 10^−4^, p = 10^−6^, p = 10^−4^, p = 10^−6^ for, respectively, session 208, 212, 214, 331 and 332, black stars). (**b**) Mean ± SEM across experiments and time window [0 100] ms of information and information gain computed with MUA as response feature and TB as state variable for basal and norepinephrine conditions. Black stars label significant difference between basal and norepinephrine conditions (one tailed t test, p = 10^−10^ and p = 10^−9^ for information and information gain). (**c**) Mean ± SEM across experiments with norepinephrine of the information with different response features (MUA, PC, CAT) computed over the [0 100] ms time window after stimulus. Different symbols indicate no significantly different means (F(12, 1287) = 34.4, p = 10^−30^, one-way between subject ANOVA followed by HSD multiple comparison test). (**d**) Mean ± SEM across experiments with norepinephrine of information gain and percentage information gain. Different symbols indicate no significantly different means of different response features (F(12, 1287) = 7.1, p = 10^−12^). Note that no significant difference was found for percentage information gain (p = 0.6). (**e**) Mean ± SEM across all experiments for MUA and PC2 as different response features of the direct information in a time window of [0 100] ms after stimulus computed before (black bars) and after discounting state variable TB from the single trial response *r*, respectively for, the full model (red bars), the multiplicative model (green bars) and the additive model (blue bars). (**f**) Mean ± SEM across experiments with norepinephrine of information gain and percentage information gain of the multiplicative model. Different black and red symbols indicate no significantly different means of information gain (F(10, 1089) = 4.3, p = 10^−6^) and percent information gain (F(10, 1089) = 2.7, p = 0.002) for different response features. In panels c,d and f the symbols { > < ^ = # V} mark data groups that have similar means (Tukey’s HSD, p < 0.05).

Cultures were treated with norepinephrine, a neuromodulator that was previously shown to reduce the burst frequency and to increase the sparse spiking activity among bursts, both *in-vivo*^18,19^ and *in-vitro*^20^. Our recordings showed that the norepinephrine did not alter significantly the spontaneous firing frequency (basal 1.06 ± 0.26 Hz versus norepinephrine 1.12 ± 0.45 Hz, p = 0.92, n = 5, Wilcoxon signed-rank test), but decreased the spontaneous network burst rate (basal 2.47 ± 0.67 burst/min, norepinephrine 2.11 ± 0.76 burst/min, p = 0.03, n = 5, Wilcoxon signed-rank test). Importantly, using norepinephrine led to a systematic decrease in the network synchrony (measured as the fraction of coincident spikes among spike trains recorded in different electrodes, see Methods) (Fig. 6a). Under norepinephrine, and when TB was used as a state variable, including state knowledge still provided a significant information gain (Fig. 6b). There were two noticeable differences in terms of stimulus information coding, however, between treated and untreated cell cultures. First, upon norepinephrine treatment, the information about the stimulus carried by responses *R* increased (Fig. 6b, left). Second, the information gain when considering also the state variables TB was reduced (Fig. 6b, right). When considering other population response features such as PCs and CAT, the stimulus information in neural responses and the information gain due to state knowledge (Fig. 6c and d) showed the same qualitative pattern across response features that we found for untreated cultures. As in untreated cultures, when using norepinephrine PC2 had the highest stimulus information, and both MUA and the first few PCs had a good information gain when considering state knowledge. Moreover, also when using norepinephrine no linear model did better than the multiplicative-only state-dependence model in leading to higher information gains after state discount (p = 0.052 and p = 0.9 for, respectively, MUA and PC2, one-way between subject ANOVA followed by Tukey’s HSD multiple comparison test). These results indicate that similar state-dependence processing principles apply to both basal and norepinephrine cultures, although the addition of norepinephrine may affect the strength of state dependence.

### A small subset of electrodes carries most of the stimulus information and of its state information gain

**Figure 7:**
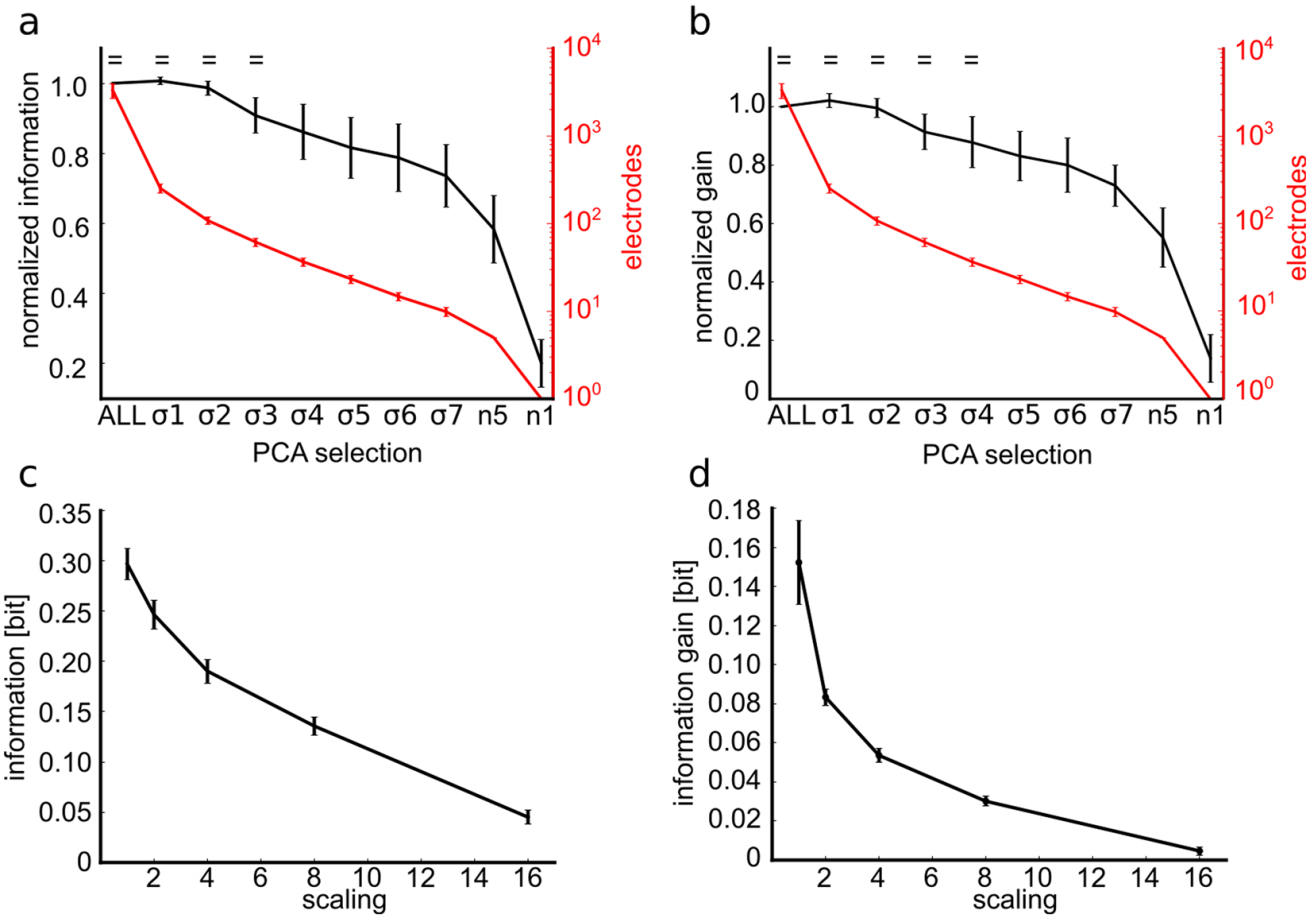
The information and information gain are carried by a subset of electrodes. For each experiment and PC, we selected subgroups of electrodes with PC weight (i.e. eigenvector values) higher than 1 to 7 standard deviations (σ1 to σ7) from the mean PC. We also selected electrodes with the highest (n1) and highest five (n5) PC weights. For any selection, the information and the information gain are averaged across PCs and experiments (n = 4) and the corresponding means and SEM are reported. (**a**) The mean information of the selected electrodes is comparable to the information of the entire electrode array (ALL) up to σ3, that corresponds to ~61 selected electrodes (F(9, 390) = 26.4, p = 10^−35^). The symbol = marks the data group that has similar means (Tukey’s HSD, p < 0.05), see Supplementary Information. (**b**) The mean information gain of the selected electrodes is comparable to the entire set of electrodes (ALL) up to σ4, that corresponds to ~37 electrodes (F(9, 390) = 20.9, p = 10^−29^). Stars indicate no significantly different means respect to ALL (one-way between subject ANOVA followed by Tukey’s HSD multiple comparison test). The symbol = marks the data group that has similar means (Tukey’s HSD, p < 0.05), see Supplementary Information. (**c**) The information decreases steeply with the downscaling of the number of recording electrodes (i.e. the scaling factors 2, 4, 8 and 16 correspond to down-sampled arrays of size 32 × 32, 16 × 16, 8 × 8 and 4 × 4, respectively). (**d**) The information gain decreases even more steeply and decays almost to half of its value with a downscaling of 2. For each scaling factor the information and the information gain are computed for different samplings of the available 64 × 64 electrodes. The mean and SEM over the different samplings are reported.

Is the response of cultured networks truly distributed across the entire network, or is there a small subset of sites that carries all or most of the information in the network? Studies from various preparations suggested that neural information is sparsely distributed across cells^21,32–34^ that is, only a small fraction of neurons genuinely contribute to information for the task at hand, so that in some cases the most informative neurons discriminate the stimuli as well as the animal does^21,35^. However, these experiments have been based only on recordings of small populations, and have not considered the distribution across large sets of simultaneously recorded cells of the state dependent gain. The large-scale recordings that we performed allowed us to assess the information carried by neural activity expressed at thousands of sites, and thus put us in a privileged position to investigate this issue. To answer this question, we compared the information carried by the whole array with information carried by considering only a smaller subset of selected electrodes. An effective way to identify channels with good stimulus-driven responses is to use the top spatial PCs, as those identify the spatial patterns with the highest variance of activity across all trials. To select the subset of electrodes, we considered the spatial map of each one of the top 10 spatial PCs, and we considered only a fraction of electrodes that had the highest weight (measured in SD units from the mean PC, see Methods). Additionally, we varied the selection threshold parametrically from 1 to 7 SD units. We then computed for each subsampled set the information it carried (averaged over all experiments and all considered top 10 PCs per experiment). Since the results obtained by thresholding of different PCs were also relatively stable, and also because the regions identified by different PCs had a relatively large overlap (Supplementary Figs S4 and S5), we averaged results obtained with different PCs. We found that choosing a threshold of 3 SD units (σ3 in Fig. 7a, which corresponded on average to selecting 78 ± 5 units per experiment, that is only a small fraction of the total of recorded channels) was enough to preserve within 95% of the total information. The information decreased when increasing the selection threshold. However, it remained not significantly different from the information carried by the whole array up to thresholds of 3 SDs. Using very high thresholds, such as 7 SDs, or even using extreme selection criteria that only considered 1 or 5 electrodes per experiment selected by the top weights (n1 and n5 respectively in Fig. 7a), still gave a surprisingly large percentage of the information computed from the whole array (e.g. 82.5% of the information is accounted for by ~24 electrodes using a threshold of 5 SDs).

We then investigated whether the gain in information after discounting state dependence, and quantified again as *I*(*S*; *R*, Θ) − *I*(*S*; *R*), is distributed across the entire population or whether it can be achieved by the same small subset of electrodes that carries a large fraction of the stimulus information. We therefore repeated the subsampling analysis described above for the information gain. Results (Fig. 7b, red line) show that the information gain of the whole network can be fully recovered from the small subset on selected electrodes that carry the most stimulus information. For example, small subsets of channels selected for PCs with a threshold of 2 SD (138 ± 9 channels per experiments) are sufficient to recover 97% of the information gain of the whole array. Given that our results show that information is carried by a small subset of neurons, we wondered whether this meant that the information about network activity could be also recovered using a less dense electrode array with fewer electrodes. To investigate this issue, we subsampled the channels using a square grid with wider spacing (2, 4, 8, 16 fold less) than the native inter-electrode separation (to simulate having a less dense array) and we computed the information about the stimulus both only considering response, *I*(*S*; *R*) or including also state dependence, *I*(*S*; *R*, *TB*). We found (Fig. 7c and d) that only part of the total network information (71%, 54%, 38%, 13% for a subsampled grid with 2, 4, 8, 16 reduction from the truly used one) could be recovered from the subsampled data. Note that we found equivalent results, for a given number of subsampled electrodes, when subsampling the data either with spatial structure described above or at random.

**Figure 8:**
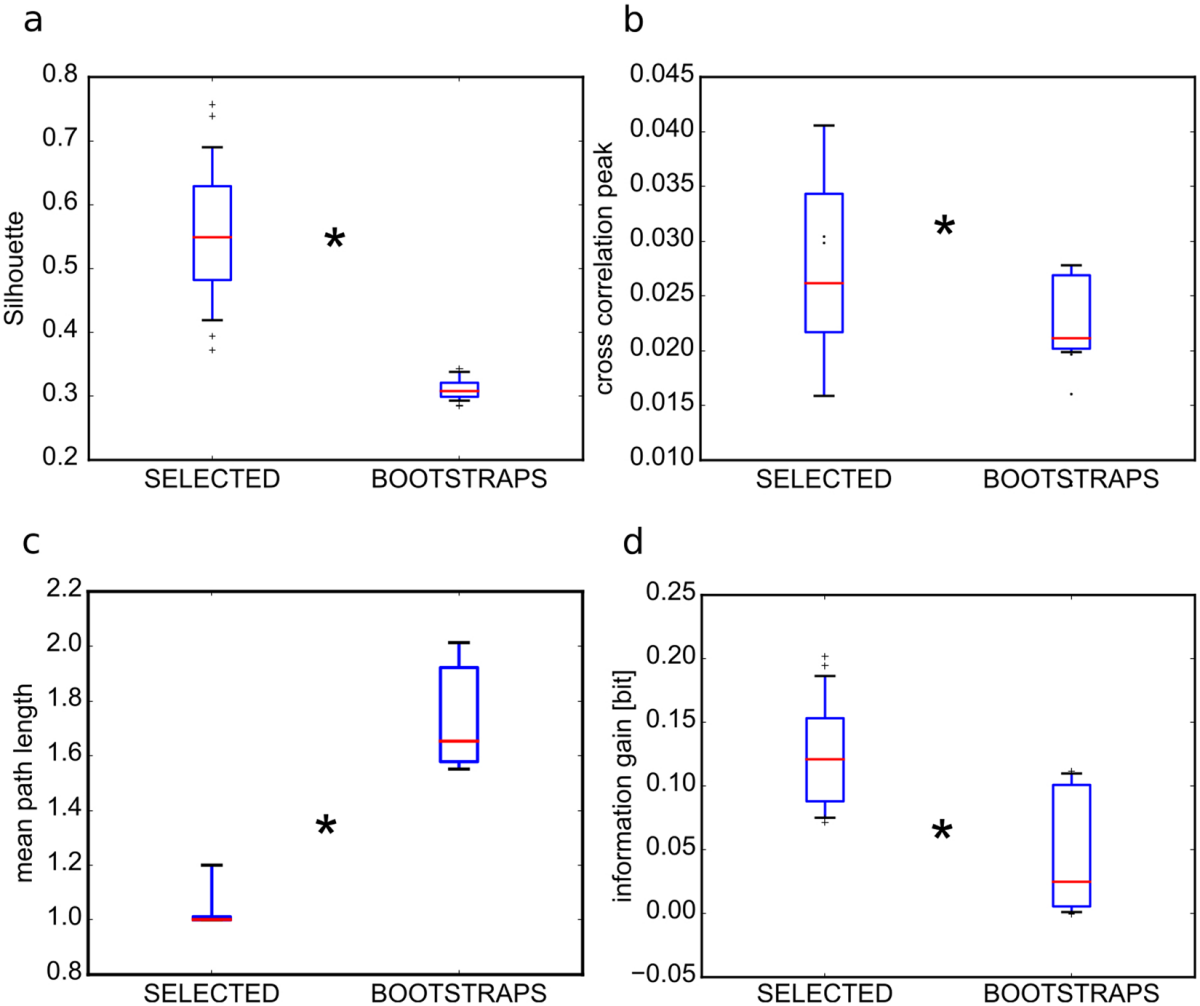
Spatial structure of electrodes selected by thresholding PCs. In all panels the electrodes are selected with PC weights higher than 3 SDs (SELECTED). All measures on SELECTED are compared to measures on equally sized sets randomly chosen from the complementary set of the non selected electrodes (n = 100 repetitions) and the 95^th^ values of those bootstraps are reported (BOOTSTRAPS). All box-plots are relative to the pooling of 10 PCs and 3 experiments. Red horizontal lines are means computed across 3 experiments, boxes show the interquartile range and the whiskers correspond to the 5^th^ and 95^th^ percentiles. (**a**) The silhouette coefficients, a measure of goodness of spatial clustering, are significantly higher when computed on the SELECTED electrodes respect to the BOOTSTRAPS (Wilcoxon signed-rank test, n = 30, p = 10^−5^). (**b**) The mean peak cross-correlation coefficients are significantly higher among the SELECTED electrodes respect to the BOOTSTRAPS (Wilcoxon signed-rank test, n = 30, p = 10^−6^). (**c**) Similarly, the mean path length is shorter among the SELECTED electrodes respect to the BOOTSTRAPS (Wilcoxon signed-rank test, n = 30, p = 0.001). (**d**) Finally, the information gain, computed over the [0 100] ms time window, is higher in the SELECTED electrodes respect to the BOOTSTRAPS (Wilcoxon signed-rank test, n = 30, p = 10^−6^).

To verify whether the selected electrodes were spatially organized in regions of the network we performed a clustering analysis on their spatial location using the DBscan algorithm (see Methods) and we quantified the goodness of the clustering procedure with the Silhouette coefficient. This analysis showed that they clustered in a few areas of the network (Supplementary Fig. S4). To assess our analysis, we also verified that the selected electrodes could be better clustered (Fig. 8a) than any equivalent random set of the same number of electrodes. We then further characterized these areas with respect to the rest of the network using graph theory measures. To this aim we first computed the strength of the functional connections using cross-correlation (see Methods). We found that the cross-correlation peaks among the selected electrodes were significantly higher than in any other size-equivalent subset of non-selected electrodes (Fig. 8b). Successively, we computed the mean-path-length (i.e. the mean of the shortest paths between any pair of electrodes) among the selected and non-selected electrodes. We found that the mean-path-length (MPL) among the selected electrodes was lower than the same measure computed over any size-equivalent subset of non-selected electrodes (Fig. 8c). As a consequence of the lower MPL, we could estimate that the connectivity among selected electrodes was much more recurrent than among subsets of non-selected electrodes. Interestingly, this is a known property that contributes to sustain state dependent processing in neural networks^1^. Seemingly we found that the information gain was significantly higher for the selected electrodes with respect to the non-selected ones (Fig. 8d).

To investigate whether the areas with highest mean firing rate correspond to the ones with the highest PC weights, we performed an additional analysis in which we quantified the overlap between groups of electrodes of increasing spontaneous firing rate and electrodes selected at different thresholds on the PC weights. Results (Supplementary Fig. S6) show that the selected electrodes with high threshold have a large overlap with the electrodes in the highest quintile of firing. Thus, areas with higher PC weights approximately, but not fully, coincide with those that fire the most. The fact that neurons firing the most carry on average more information is well documented in cortical recordings (see e.g.^21^). Here, since we could not measure local cell density in the cultures from which we recorded neurophysiological responses, we could not determine if the electrodes with more information and firing corresponded to regions with higher cell density or higher excitability.

In sum, the implemented selection criteria allowed the determination of a subset of electrodes that are informative as the whole set of electrodes about external stimuli; that cluster in few regions of the network in which activities are efficiently broadcast and where most of the state dependent processing takes place.

## Discussion

The ability of networks of neurons to process external stimuli is not a fixed property, but it can vary from time to time to match changes in the operational demands^36^. These changes in operational modes are under the control of various factors, which include neuromodulation^37–39^, spontaneous changes in network excitability and others. These changes are believed to profoundly affect stimulus-response relationships, for example enhancing or suppressing the stimulus-response gain or changing the threshold at which weaker stimuli elicit a strong enough response to be detected. Signatures of changes in the internal state of the network can be in part detected through changes in the spontaneous activity of the network prior to stimulation. However, studies of how state changes, as revealed by spontaneous activity, affect stimulus-response representations have been limited mainly to single cells or small populations of neurons^4,6,40^. Here we built on these previous studies by considering how different features of population spontaneous dynamics affect the responses of very large populations of neurons. We took advantage of the unique sampling of neural activity offered by *in-vitro* networks grown on high-resolution CMOS-MEA devices and capable of large-scale recordings and on-chip electrical stimulation. Neuronal cultures respond effectively to stimuli^26,41^ and have a rich structure of spontaneous network bursting activity^42^ and therefore are ideally suited to this study. The implications of our results are discussed in what follows.

### Advances in characterizing state dependence in large scale networks

Previous studies have highlighted how the responses of relatively small neural populations depend on the spontaneous activity prior to stimulus presentation^43–45^ and how the state dependence of these responses to stimuli can be used to better extract information from neural activity^4^. In particular, previous studies of our group^4,31^ have proposed that predicting with a model of state dependence the fluctuations of the single trial response around its mean in each trial can drastically improve decoding of population activity. This is because this calculation allows the identification and elimination of network-level sources of variability that, being correlated across neurons, cannot be eliminated and averaged away just by increasing the number of electrodes and averaging over more neurons. One of the main advances we made in this study is to characterize state dependence and information gain due to state knowledge in large neural populations densely sampled with thousands of electrodes. Demonstrating that we can gain information from large networks with knowledge of pre-stimulus state is an important step for proposing the viability of state-dependent coding as a coding mechanism in the nervous system. In fact, it could be that we report a state information increase at the level of single neurons or few neurons but perhaps this gain would be absent if we considered a larger network, because the information that is only available in state for one neuron may be available as post-stimulus response in another neuron. The fact that we find still a sizeable advantage in very large networks suggests that the above scenario can be ruled out, and adds strong support to the relevance of state dependent information. It also supports the idea that this state dependent variability reflects variability shared at the network level which cannot be removed by adding more electrodes (because, being common or shared across electrodes, it cannot be averaged away by considering more electrodes). This study therefore strongly suggests that implementing state dependent decoding rules in decoding of brain activity can improve the performance of brain machine interfaces (BMI), even when those are based on large numbers of recording electrodes. The difficulty of increasing the performance of BMIs by increasing the number of electrodes has been recognized as a main bottleneck of BMI developments^46–48^. Our study suggests that the use of state dependent decoding algorithms can ameliorate this problem.

The access to a large fraction of neurons in the network also allowed us to probe the sparse nature of this information encoding. Previous studies based on simultaneous recordings of a small number of neurons reported that most of the population information is carried by a few informative neurons^21^ and that the most informative neurons carry nearly enough information to support the discrimination abilities of the whole animal^35^. Our results show that this picture, that was formed over the years from small population recordings, also holds for large-scale recordings. In particular, our work shows that one main advantage of recording from a large number of neurons in the network is that it facilitates the individuation of the neurons that carry the most stimulus information. Our finding that the information gain found at the whole network level was also found when considering the few selected neurons that carry the core of the network information again suggests the importance of considering state-dependent coding. This is compatible with the view put forward above that state dependent coding is an effective way to get rid of part of the variability shared by all neurons in the network, including the most informative ones.

### Importance of state dependence for population coding

The presence of state-dependence puts profound – but still largely unexplored – constraints on how population codes operate^14^. State-dependence may imply that populations transmit information only using codes that are robust to state fluctuations. Alternatively, downstream areas may extract variables indicating the current state from network activity and then use state-dependent decoders to interpret population activity. The information theoretic formalism used here allows to inform us quantitatively on how efficient the two above population codes may be^4,40^. A high value of stimulus information from neural responses *I*(*S*; *R*) obtained without knowledge of network state would support the first scheme, that of using state-independent variables for coding and transmitting information^40^. On the other hand, a lower value of *I*(*S*; *R*) paired with a higher value of information in neural response *I*(*S*; *R*, Θ) obtained when including knowledge of state would support the latter coding scheme^4^. The fact we found in all datasets more information when including knowledge of state dependence suggests that it would be more efficient to pass information to other networks through a state dependent code. How and if this may happen remains to be investigated. However, given that both state variables and network responses computed here were based on spiking activity, they are in principle accessible to downstream networks.

In networks recorded *in-vivo* from awake behaving subjects, a potential way to investigate whether the network reads out state dependent coding information is to measure not only how much stimulus information is gained by knowledge of the state dependence, but also to measure, using the concept of intersection information, how much of this information gain is turned into behavioural performance^49,50^. When recording simultaneously from several different networks, whether or not the state dependence is used to pass information form one network to the next can be measured, using similar concepts, by measuring if state dependent codes in one network influence responses in a downstream network in the same trial. Encouraged by the results of the present work that state dependent coding advantages exist also in largely sampled networks of thousands of neurons, we plan to verify these hypotheses in future experiments with dense electrode arrays *in-vivo*.

### Changes of state-dependent information with the level of network synchronization

In our experiments, we manipulated the level of network synchronization by using norepinephrine. In cultures with norepinephrine, we found that overall lower synchronization was accompanied by an increase of information in state-independent codes and a decrease of information gain when considering state-dependent codes. This result is again fully consistent with our view that considering the state dependence of neural responses is an effective way to reduce the variability that is shared across all electrodes and that cannot thus be eliminated by sampling more neurons to better average away this variability (i.e. synchronized network, by definition, have more shared variability). Previous experimental investigations^51^ have shown that norepinephrine increases the strength of inhibitory connections in the cortex. Our results on changes of information processing when applying norepinephrine drug are thus compatible with a previous computational study that investigated the role of interneuron mediated synchrony in information processing^52^. This work proposed that when interneurons have a strong enough effect to generate asynchronous states, an external input elicits a highly reliable response (i.e. high stimulus related information). In contrast, in a more synchronous firing regime, strong fluctuations of spiking activity tightly interact with delivered stimuli allowing state dependent processing of information. All in all, these results suggest that state dependent coding mechanisms may be used and be more crucial in synchronized networks, as it is an effective coding mechanism to transmit information robustly despite the shared variability of neurons in these networks.

### Network bursts as effective state parameters

Our data show that cultured networks prepared from embryonic hippocampal neurons express variable responses to electrical stimuli that are strongly modulated by the time interval (TB) between the stimulus and the last spontaneous network burst. This time interval was thus considered to be the most effective state variable. Additionally, we found that this state variable acts largely by modulating the stimulus-response gain of the network. Previous studies^11,13,53,54^ on cultured networks also described the existence of relationships between network response features and the stimulus latency relative to the previous burst. A previous study^54^ showed that responses of cultures to a given stimulus and recorded with a 64 electrode array are weaker (respectively stronger) at shorter (respectively longer) TB values. Our results extend this previous result to study the modulatory effects of TB on the network response, sampled with thousands of electrodes, by a set of different electrical stimuli. The consideration of multi-stimuli responses was essential for us to show that the modulation of TB acted as a multiplicative modulation term of the stimulus-response gain, rather than as an additive term. The fact that we could well fit state dependence with a simple multiplicative model opens up the possibility to use our new results on the dependence of network responses on TB for online control of single site stimulation^54^ to the control of multiple-site stimulation, thus opening up the possibility to acquire hints for developing better bidirectional control of BMIs. Previous results^11,13^ have also suggested that the occurrence of a spontaneous burst wipes out the information dependence of the post-burst response on the previous stimulus.

Our results suggest that refractory effects following a network burst may be responsible for the state modulation of population responses to stimuli. It is important to note that this kind of state-dependent modulation, entirely based on features of the ongoing activity before stimulus presentation, differs substantially from the kind of state dependence, mostly related to behavioural state changes such as attention, arousal and network synchronisation, implied by most studies of state-dependence in cortex^10^. However, one possible link between the results obtained here and the periodic gain rescaling observed *in vivo* in cortex at different phases of the theta rhythm^6^ is that resets of cortical theta phases are often preceded by bursts of network depolarization of duration similar to the network bursts documented here^55^. This suggests that, while our notion of *in-vitro* state dependence differs from the one usually employed in cortical studies, some of the mechanisms observed here may relate to some of the effects of ongoing pre-stimulus activity on cortical stimulus-response relationships.

### Possible mechanisms for gain rescaling

We found that the main effect of the time between the last spontaneous burst and the stimulus was to rescale the gain of network response to stimuli, with the network eliciting weaker responses from shorter times from the last burst. This effect on the gain may arise from several possible mechanisms. The increase of network responses at longer TB may be mediated by asynchronous synaptic release and spontaneous excitatory post-synaptic currents increase when the evoked synchronous release is depressed^56,57^. Or following a network burst the neuronal culture enters into a refractory period. Further, shortly following a network burst the synaptic connections may be depressed because of synaptic depletion^58,59^ and, consequently, neurons may respond more weakly to stimuli.

### Spatial structure of the most informative regions

Finally, by analysing the graph network properties we systematically found that the electrodes carrying the most information had a specific spatial organization. These informative electrodes were grouped in regions of the network and were characterized by strong functional connections with low average path lengths. Previous studies *in-vivo*^60,61^ and *in-vitro*^62^ showed that neural networks are characterized by a small-world topology, with a short mean path length and a high clustering coefficient. Moreover, these neural networks are typically characterized by the presence of hubs, or groups of neurons with a high out/in degree that allows relaying information quite effectively to a large portion of the network. This property likely plays a special role in information processing. Recently, it has also been shown in cell cultures^63^ that hubs might be involved in broadcasting spontaneous activity from early-to-fire neurons to the whole network. Additionally, we recently showed, in a computational model^23^ validated against our high-resolution electrode array recordings, that such ‘functional hubs’ (called functional communities in^23^) can naturally emerge in random networks in which the degree of connectivity is comparable to cell cultures and the probability of connection decays with the interneuron distance. Here, we proved the presence in these networks of spatially organized subsets of more informative neurons. Taken together, these facts suggest that, even if neurons are plated homogeneously, they can self-organize to generate small subsets of neurons that, due to their highly organized spatial structure, their graph theoretic properties, and strong connectivity, may act as hubs able to broadcast both state dependent and state independent information.

## Materials and Methods

### Ethical statement

All procedures involving experimental animals were performed in accordance with the Italian and European Union guidelines and regulations. All animal procedures carried out in this work were approved by the institutional Istituto Italiano di Tecnologia (IIT) Ethics Committee and by the Italian Ministry of Health and Animal Care (Authorization number 110/2014-PR, December 19, 2014). The primary rat hippocampal cultures were obtained following procedures described in Supplementary Methods and in previous work^64^.

### Data availability statement

All data can be downloaded from http://www.sicode.eu. All scripts used to analyze the data will be provided upon request to the lead contact author.

### Quantification of the state variables

We investigated a set of potential state variables θ, all obtained from spontaneous activity recorded in the last 4 seconds preceding the stimulation. Most such variables were defined in terms of the network burst (NB)^23,27^. A NB was identified when the pooled network activity, binned in 20-ms bins, exceeded a threshold T. The threshold T was determined as 10% of the maximal binned spike count in a session. The timing of the NB was refined by sliding (slid by 1 ms) leftward the bins until the binned activity fell below threshold.

We considered the following as candidate state variables: the time interval between the stimulus and the last network burst before stimulation (TB), the number of spikes in the last network burst (NSP), the ignition site of the last network burst (IS), the network burst rate (NBR, the number of NB) and the mean firing rate (MFR, the mean number of spikes per electrode divided by the recording time window). We also considered the phase at stimulus time and the pre-stimulus time-averaged amplitude of the network multi-unit activity (MUA, see Supplementary Information), computed with the Hilbert transform of filtered in 6 different frequency bands ([1 6], [6 12], [12 18], [18 30], [30 50] and [50 100] Hz) using a least-square finite impulse response filter with 1-Hz transition bandwidth.

### Mutual information

To quantify stimulus coding we used mutual information measures. First, we computed the information *I*(*S*; *R*), about which stimulus s (out of a set *S* of possible stimuli) was being presented in a given trial, and the post-stimulus response *r* (out of a set *R* of possible responses) in the same trial. Second, we quantified the information *I*(*S*; *R*, Θ) about the stimulus carried by the joint observation, in the same trial, of the post-stimulus response *r* and the pre-stimulus state parameter θ. These quantities were defined as follows:

3 $$I(S;R)=\sum _{s,r}P(s)P(r|s){\mathrm{log}}_{2}\frac{P(r|s)}{P(r)}\,$$

4 $$I(S;R,{\rm{\Theta }})=\sum _{s,r}P(r,\theta |s){\mathrm{log}}_{2}\frac{P(r,\theta |s)}{P(r,\theta )}$$

where *P*(*s*) is the probability of presentation of stimulus *s*, *P*(*r*) is the probability of observing *r*esponse *r* across all trials to any stimulus, *P*(*r*|*s*) and *P*(*r*, *θ*|*s*) are the probability of observing response *r* given the presentation of stimulus *s* and of observing response *r* and state θ, in the same trial, given pre*s*entation of stimulus *s*, respectively. Information is measured in bits (1 bit corresponds to a reduction of uncertainty by a factor of two). Details of the direct numerical calculation of information from the above equations is given in Supplementary Information, Section “Numerical Procedures to compute direct estimates of mutual information”.

We defined the information gain due to the knowledge of state as: *I*(*S*; *R*, Θ) − *I*(*S*; *R*). This measure has the advantage of concentrating the effect of *θ* on the stimulus dependence of r (see Supplementary Methods, section “Information Gain”, for more details).

### Linear model of the dependence of the single trial response on the state variables

We modelled the single trial neural response *r* as a function of the state variable θ with θ = TB. As a first step, we checked whether TB affects *r* as an additive term to the mean response at fixed stimulus 〈*r*_*s*_〉. To this end we computed *dr* as the average across all stimuli of the trial-to-trial response variability at fixed stimulus: *dr*(*TB*) = 〈*dr*〉_*S*_ = 〈*r* − 〈*r*〉_*S*_〉_*S*_ and evaluated how *dr* depends on TB. We also checked whether the state variable TB affected the mean response to the stimulus 〈*r*_*s*_〉 by estimating how the ratio *r*/〈*r*_*s*_〉 depends on TB. Finally, we estimated *r*_*model*_ as the sum of two components, the first one takes into account the multiplicative term that scales the population response at fixed stimulus while the second one takes into account the additive offset that affects all trials at a given state variable TB:

5 $${r}_{model}=g(TB){\langle r\rangle }_{{\bf{S}}}+b(TB)$$

where we evaluated the slope *g*(*TB*) and the additive *b*(*TB*) functions, of each experiment and at each *t*ime point *t* after the stimulus, with a least square solution of the equation *r* = *g*(*TB*)〈*r*_***S***_〉 + *b*(*TB*) at fixed TB, with TB discretized in 6 values.

For each experiment we fitted the functions *g*(*TB*, *t*) and *b*(*TB*, *t*):

6 $$\begin{array}{rcl}g(TB,t) & = & k(t)[1-{e}^{\frac{-(TB)}{\tau (t)}}]\\ b(TB,t) & = & A(t){e}^{B(t)TB}+C(t){e}^{D(t)TB}\end{array}$$

Together with the full model we also considered the purely multiplicative (*b*(*TB*) = 0) and additive models (*g*(*TB*) = 1). Then, for each time point *t* we discounted trial-to-trial state-dependent response variability from single-trial response by Eq. (7):

7 $${r}_{d}=r-dr=\frac{r-b(TB)}{g(TB)}$$

The evaluation of Eq. (7) is problematic for small TB (i.e. small *g*(*TB*), 15 to 20% of the total trials). The latter trials (say M) were removed before computing *I*(*S*; *R*_*d*_). In order to compare it with the other information measures, we randomly removed M trials from the calculation of *I*(*S*; *R*), *I*(*S*; *R*, Θ) and *I*(*S*; *R*, Θ_SH_) with Θ = TB. We repeated the random selection procedure 10 times and we took the average information. The trials removed from *I*(*S*; *R*), *I*(*S*; *R*, Θ) and I(*S*; *R*, Θ_SH_) are not the same ones removed from *I*(*S*; *R*_*d*_), to avoid any knowledge about Θ to the responses *R*. We modelled *r*_*model*_ with the “full model”, which includes a slope and a background component, as well as the contribution of the two parts, namely the “purely multiplicative” model (*r*_*model*_ = *g*(*TB*)〈*r*_*S*_〉) and the “purely additive” model *r*_*model*_ = 〈*r*_*S*_〉 + *b*(*TB*). We evaluated the goodness of fit of all models with the coefficient of determination ${R}_{adj}^{2}(t)$ between response set *R* and *R*_*model*_ at fixed stimulus and for each time, defined as:

8 $${R}_{adj}^{2}=1-(\frac{n-1}{n-k})\frac{SSE}{SST}$$

where *SSE* is the sum of squared error, *SST* is the total sum of squares, *n* is the number of trials and *k* is the number of model parameters. We also compared the performance of each model by using the Bayesian information criterion (BIC):

9 $$BIC=n\,{ln}(\frac{SSE}{n})+k\,{ln}\,n$$

For each model *j* we computed the BIC weight:

10 $${w}_{j}=\frac{{e}^{(BI{C}_{min}-BI{C}_{j})}}{{\sum }_{j}{e}^{(BI{C}_{min}-BI{C}_{j})}}$$

A given weight yields the evidence in favour of model j being the actual best model among the considered set of models.

### Electrode selection criteria and clustering

We performed an analysis to determine if information and state dependency could be equally well explained when considering a subset of highly representative electrodes over the 4096 array. For selecting the electrodes, we used Principal Component Analysis. In particular, each eigenvector *PC*_*j*_ (with *j* = 1, …, 10) allows to associate a weight to all electrodes: $P{C}_{j}=[P{C}_{j}^{1},P{C}_{j}^{2},\ldots ,P{C}_{j}^{4096}]$. We define *μ*_*j*_ and *σ*_*j*_ as, respectively, the mean and the standard deviation across the *PC*_*j*_ weights and we select the electrodes *i* satisfying the criteria $|P{C}_{j}^{i}-{\mu }_{j}| > K{\sigma }_{j}$. The parameter *K* was swept over the interval [1 7] and regulates the strength of the selection. We also performed a selection on the electrodes that fell more apart from the mean value *μ*_*j*_ and considered the most (n1) and the five most significant electrodes (n5). The overlap between sets A and B was quantified as 100 · #(A ∩ B)/min(#A, #B), where ∩ is the intersection and # indicates the cardinality of the corresponding sets. The overlap measure ranges from 0 (no intersection) to 100 (full intersection). The significance of the intersection (p < 0.05) between different sets of electrodes was assessed with the hyper-geometric test. The clustering of the selected electrodes was performed with the density based DBscan algorithm and its parameters were determined by maximizing the Silhouette coefficient. In order to assess the significance of the Silhouette coefficient we considered randomized equivalent sets of electrodes (n = 100 repetitions, same number of electrodes of the original set) and computed the 95^th^percentile of the bootstrapped silhouette coefficients. The clusters were visualized in terms of alpha shapes of parameter α = 0.1 (Supplementary Fig. S4).

## Electronic supplementary material

Supplementary Information
