# Supplementary material for: State-dependent representation of stimulus-evoked activity in high-density recordings of neural cultures
Source: Sci Rep. 2018 Apr 3;8:5578. doi: 10.1038/s41598-018-23853-x (PMC5882875; doi:10.1038/s41598-018-23853-x)
Supplement: Supplementary file 1 — Supplementary Information [file 41598_2018_23853_MOESM1_ESM.pdf]

Supplementary Information to “State-dependent representation of stimulus-evoked activity in high-density recordings of neural cultures”

Thierry Nieus[1,4*¶], Valeria D’Andrea[2¶], Hayder Amin[1], Stefano di Marco[1#a], Houman Safaai[2,3], Alessandro Maccione[1], Luca Berdondini[1&], Stefano Panzeri[2&*]

[1] NetS[3] Laboratory, Neuroscience and Brain Technologies Department., Istituto Italiano di Tecnologia, Genova, Italy.

[2] Neural Computation Laboratory, Center for Neuroscience and Cognitive Systems @UniTn, Istituto Italiano di Tecnologia, Rovereto, Italy.

[3] Department of Neurobiology, Harvard Medical School, 02115 Boston, MA

[4] Department of Biomedical and Clinical Sciences "Luigi Sacco", Università di Milano, Milano, Italy

[#a] Current Address: Department Scienze Cliniche Applicate e Biotecnologiche, Università dell’Aquila, Italy

*Corresponding authors

E-mails: thierry.nieus@unimi.it (TN) (lead contact), stefano.panzeri@iit.it (SP)

[¶] TN and VD are Joint First Authors.

[&] SP and LB are Joint Senior Authors

Supplementary Methods

Preparation of neuronal cultures and CMOS-MEA recordings

We performed electrophysiological measures of ongoing and electrically evoked responses from cultured neuronal networks with primary neurons grown on high-density CMOS-MEAs (BioChip 4096E, from 3Brain AG, Switzerland), that provide up to 16 on-chip stimulation sites and 4096 recording microelectrodes, both of 21 μm x 21 μm in size. We prepared primary hippocampal neurons from E18 rat embryos (Sprague Dawley from Charles River Laboratories International) [1]. Before cellular seeding, we prepared chips as follow. First, we sterilized the outside of the chips with a tissue moistened with EtOH 96%. Then, we sterilized the well of each device for 20 min. with 70% EtOH and we rinsed abundantly with double-distilled water (DDW) before drying. We pre-conditioned the devices overnight in an incubator with the well filled with Complete Neurobasal Medium, containing 2% B-27 1% penicillin/streptomycin and 1% GlutaMax supplements (all reagents from Life Technologies). Successively, we prepared the active area of each device for cellular seeding by coating the electrode array area with 50 μg/ml poly-dl-ornithine (PDLO) (Sigma-Aldrich) as adhesion promoting layer and incubated overnight at 37°C and 5% CO_2. The day after, we rinsed the CMOS-MEAs 3 to 4 times with sterile DDW and we left them drying under a sterile hood. We seeded cells on the pre-coated substrates and we maintained them in an incubator at 37°C with 5% CO_2 and 95% humidity. To do so, we diluted the cellular suspension in medium (Neurobasal, from Invitrogen) at a nominal concentration of 1000 cell/μL and we seeded drops of 90 μL on the active area of the chips. After 2 hours from cellular seeding, we added Neurobasal supplemented with 2% of B27, 1% of Glutamax. This resulted in a neuronal seeding density of ~2500 cell/mm^2. The medium was exchanged every four days by removing 1/3 of the total volume and by replacing it with the fresh complete Neurobasal medium. Experiments started after 24 days-in-vitro (DIVs) cell culture. To record and evoke spiking activity from CMOS-MEAs we used a custom setup (sampling frequency of 7.7 KHz/electrode for full-array recordings) connected to a Plexon Stimulator 2.0 (Plexon Inc., USA). The experimental protocol included three phases that consisted in the recording of the spontaneous activity before electrical stimulation (10 min), the

recording of the ongoing activity and electrically evoked responses (40 min., 60 trials per electrode) and the recording of the post-stimulation activity (10 min.). The same protocol was repeated after addition of norepinephrine (5 μM , 10 min. incubation). We used a PDMS cap to ensure sterile and stable conditions in the cell culture well during long experimental recordings. For the electrical stimulation we applied rectangular biphasic current pulses (amplitude adjusted between 200-400 μA; duration of 600 μsec). The network was stimulated at 0.2 Hz, i.e. inter-stimulus was 5 seconds, and each stimulus was delivered from 8 spatially distinct sites using randomized electrode sequences. To program the stimulation sequences and to operate the electrical stimulator we developed a software tool in C# language (Visual Studio 2010; .NET framework 3.5).

Spike detection and first order statistics of network activity

We performed spike detection by using the BrainWave software (3Brain AG, Switzerland) and the Precise Timing Spike Detection (PTSD) algorithm [2]. Successively, we computed mean activity parameters to characterize the network activity. We performed the first order statistics and network burst detection as previously reported [3]. This includes the computation of the mean firing rate (i.e. total number of spikes over the recording duration), the mean bursting rate (i.e. total number of burst over the recording duration), the network burst rate and the mean intra-burst firing rate (i.e. the number of spikes fired in the network burst).

Quantification of the response features

We quantified the stimulus-evoked response of the network in terms of different features of the spiking activity, computed as follows. First, for each electrode, the spiking activity was smoothed by counting the spikes in temporal windows of fixed duration (i.e. 20 ms) and slid by 5 ms over the time window of interest. This procedure yielded the spike count: $ST(t) = [st_1(t), st_2(t), \dots, st_k(t), \dots st_N(t)]$, where $st_k(t)$ is the number of spikes elicited by the electrode k ($k = 1, \dots, N$; with N=4096) in the time window $[\mathrm{t} \quad \mathrm{t}+20]$ ms. Note that the first 10 ms time window after the stimulus, the so called “early phase” of the response, was removed since in this time interval the response is very reproducible across trials because it does not involve synaptic

transmission [4,5] and state dependent processing likely does not take place in this early response. The network spike count (MUA) was computed as the sum of $ST(t)$ across all electrodes. We further analysed the spike count $ST(t)$ in terms of the centre activity trajectory (CAT, see [6]) and principal component analysis (PCA). With the CAT, the N-dimensional network activity is reduced to the centre of mass of the spiking activity (i.e. two coordinates) over time bins of 20 ms and it provides a representation of the propagating network events. With the PCA, the variance of the N-dimensional network activity is represented in a lower dimensional space and it has proven to provide an effective mean to cluster propagating activities in cell cultures [7]. In the latter lower dimensional space the spike count $ST(t)$ can be approximated by $\sum_{j=1}^{M} \alpha_j(t) \cdot PC_j$ where $\alpha_j(t) =< ST(t), PC_j >$ is the projection of $ST(t)$ onto the eigenvector (basis) PC_j and M is the dimension of the lower dimensional space ($M<<N$). Examples of spatial representations of the PCj are reported in Supplementary Fig. S4 (j=1,2,3) while the time course of the different PCs used to compute information is represented by the $\alpha_j(t)$ functions.

Numerical procedures to compute direct estimates of mutual information and their statistics

Here we detail the numerical procedures we used to compute direct mutual information estimates from Eqs (3-4) of the main text.

We first facilitated the sampling of probability by considering a low-dimensional representation of r and θ, by discretizing r and θ in six equi-populated bins. The response could be either the MUA, the CAT or a PC score at any given time point in a [0 500] ms post-stimulus time window. The state variable θ was the time TB between last burst and stimulus application.

The estimation of the information from probabilities computed from a limited number of trials suffers from a systematic error[8]. To correct for this problem, we used the Panzeri-Treves [9] correction that subtracts the bias of each information quantity.

In the following we explain how we evaluated with non-parametric permutation statistics, if a state variable significantly changes the stimulus dependence of neural responses. This happens if the

state dependent information $I(S;R,\Theta)$ is larger than $I(S;R)$. However, when comparing these measures we had to consider that $I(S;R)$ has a smaller sampling bias than $I(S;R,\Theta)$ because the space of possible neural responses to a stimulus is one-dimensional rather than two-dimensional [8,9]. To avoid this problem that may bias the comparisons, we first estimated $I(S;R)$ as $I(S;R,\Theta_{SH})$, that is a measure of information about the stimuli obtained jointly from responses and states, but after randomly permuting the values of the state variables Θ across trials at fixed value of response variables R. This shuffling of Θ ensures that the quantity $I(S;R,\Theta_{SH})$ has the same asymptotic value as $I(S;R)$, for infinite number of trials, but has the same bias as $I(S;R,\Theta_{SH})$ for a finite number of trials. We repeated the procedure 200 times and we used the distribution of random shuffles as a null hypothesis distribution and we computed $I(S;R)$ as the 50th percentile of shuffled distribution $I(S;R,\Theta_{SH})$. The ranking of $I(S;R,\Theta)$ among the shuffled test statistic $I(S;R,\Theta_{SH})$ gives the p value of the null test hypothesis of no difference between $I(S;R,\Theta)$ and $I(S;R)$. When conducting multiple comparisons (that is, for example, when considering multiple time points) we corrected for multiple comparisons using a False Discovery Rate (FDR) [10].

Computation of information from the confusion matrix of a decoder

When including several state variables, the direct calculation of information from the stimulus-state-response probabilities (as in Eqs 3,4 of the main text) became problematic because of limited sampling issues. In this case, we thus computed information through a decoding approach [9,11]. For each trial, we decoded the predicted stimulus sp from the neural activity recorded in that trial (see below for details). From these results, we then computed the confusion matrix $Q(S,SP)$ of the joint probability of presenting stimulus s and decoding stimulus sp. Finally, we computed the information obtained through the decoder as the information contained in the confusion matrix [11], as follows:

$$\sum_{s,sp} q(s,sp) \log_2 \frac{q(s,sp)}{p(s)q(sp)} \qquad \text{(S1)}$$

The decoding was performed using a linear discriminant analysis (LDA) classifier, with a 5 fold cross-validation to separate training data (those used to set the boundaries of the linear discriminant) and test data (those used to decode stimuli and compute the confusion matrix). For computation of information decoded from the neural responses alone, we used a one-dimensional neural activity feature consisting of the binned state post-stimulus responses (6 bins). For computation of information decoded from the neural responses and state variables, we used a multidimensional neural activity feature consisting of the binned post-stimulus responses and the considered state variables (6 bins for each feature). For consistency, the binning of the neural activity features into 6 bins was the same applied for the direct information calculation (Eqs 3-4 of the main text).

We corrected the bias of the decoded information with the same Panzeri-Treves [9] procedure used for the direct information estimates of Eqs. (3,4) of the main text. To evaluate the significance of the increase of information due to state dependence, we used for the decoded information the same non-parametric permutation used for the direct information and detailed above.

The advantage of computing information through the decoding matrix is that this approach is data robust [11]. However, the information in the decoder's confusion matrix, Supplementary Information Eq. S1, is a lower bound to the total information in neural activity, which is quantified by the direct information, Eqs 3-4 of main text [11]. In other words, the information computed through a decoder cannot be higher, and is in general much less, than the total information in neural activity. As detailed in [11], the information loss when decoding can be due to either or both of two factors. The first is the potential inaccuracy of the decoding model in describing the relationship between neural activity and stimuli. For example, a non-linearity in this relationship cannot be fully captured by a linear decoder. The second is that reporting only the identity of the most likely stimulus given neural activity, as the decoder does, leads inevitably to an intrinsic loss of some of the available information. For example, reporting only the most likely stimulus loses information about the relative likelihood of each stimulus that is present in neural activity. Comparisons of information quantities computed with the direct computation of information and through a decoder can be used

to evaluate the efficiency of a neural decoding algorithm in extracting information from neural activity.

Information gain

We defined the information gain due to the knowledge of state as the difference $I(S;R,\Theta) - I(S;R)$. This measure has the advantage of concentrating the effect of θ on the stimulus dependence of r and this can be appreciated by considering that the following information-theoretic equality holds [12] :

$$I(S;R,\Theta) - I(S;R) - I(S;\Theta) = \langle I(R;\Theta|s)\rangle_s - I(R;\Theta) \quad \text{(S2)}$$

where $\langle I(R;\Theta|s)\rangle_s$ is the mutual information between R and Θ given the stimulus s. Making the reasonable hypothesis that $I(S;\Theta) \cong 0$ (that is verified in our data, see Supplementary Fig. S1) it follows that, if the relationship between response r and state θ depends on the stimulus, the difference $I(S;R,\Theta) - I(S;R)$ will be positive, and it will be zero otherwise. Hence difference between $I(S;R,\Theta)$ and $I(S;R)$ captures the effect of state on stimulus-dependent response relationships. We also defined the percentage information gain as $100 * [I(S;R,\Theta) - I(S;R)]/I(S;R)$ and we evaluated the amount of information explained by the linear model by computing the discount information ratio as: $I(S;R_d)/I(S;R,\Theta)$, where R_d are the responses after discounting the predicted single-trial variability due to state dependency .

Spike synchronization

We quantified the synchrony of the spontaneous spiking activity and how it changes when norepinephrine is added to cell cultures. To this aim we analyzed the spontaneous activity when no stimulus was applied to the cultures and we used the software SPIKY [13] to compute the synchronization coefficient. Given the large number of electrodes of CMOS-MEAs, we sub-sampled the electrode array and computed the synchronization coefficient on N=100 electrodes randomly selected from the whole array. The random selection was repeated 100 times and the

synchronization coefficient was estimated as the average across all repetitions. The synchronization coefficient computed by the SPIKY software was obtained by averaging over the whole recording duration the time-resolved measure called SPIKE-synchronization. The latter is a measure of similarity between simultaneously recorded spike trains that quantifies the fraction of coincident spikes among all spike trains. SPIKE-synchronization values range between zero (spike trains do not contain any coincidences in a small time window) and one (each spike in every spike train has one matching spike with the other spike trains).

Functional connectivity analysis and properties of the graphs

We performed a cross-correlation analysis [14] to quantify the strength (i.e. cross-correlation peak) of the functional connections (FC) between all pairs of electrodes. In order to retain significant functional links, we introduced a null model obtained by shuffling the original spike trains while maintaining the original ISI distribution. This procedure was applied to each pair of spike trains (n=200 repetitions) and the 95th percentile of the shuffled distribution was used to define a threshold ($CC_{95\text{-}sh}$) of acceptance for the functional links (i.e. only the links with $CC > CC_{95\text{-}sh}$ were retained for further analysis). From the significant links, we built a functional graph and we quantified the mean path length (i.e. mean of the shortest paths between pairs of electrodes, MPL). Importantly, in the calculation of the MPL among electrodes belonging to a certain group we allowed the paths to be constituted of electrodes falling outside the specified group. For instance, paths among electrodes of a given cluster could extend beyond the cluster itself.

Conventions for plotting the results of Tukey's HSD multiple comparison's test.

In Fig. 2b, 3a-b, 5b-c, 6c-d-e-f and 7a-b the one-way ANOVA test was used to assess significant differences ($p<0.05$) when two or more groups were compared. The ANOVA was then followed by Tukey's HSD multiple comparison test to determine which group of data did not show significantly different means among groups. The symbols {> < ^ v = - # } were used to mark the data groups (among those listed on the x axis) that have similar means between groups, according to Tukey's

HSD multiple comparison test, p< 0.05. For example, in Fig.6C the symbol > indicates that the mean information across PC2 and PC3 is not significantly different, and the symbol < indicates that the mean information across PC3, PC4, PC5, PC6, PC7 and CAT is not significantly different, etc.

Supplementary Figures

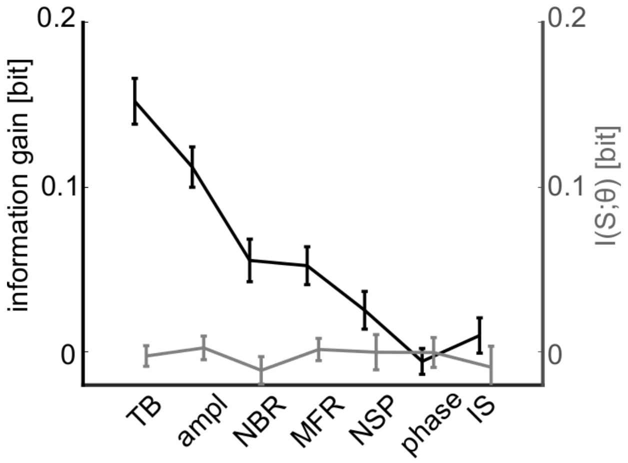

Supplementary Figure S1. The pre-stimulus state variables Θ carry information about stimuli only through the synergistic interaction with the post-stimulus responses R. None of the tested state variables carry *per se* stimulus information ($I(S;\Theta)$, grey line). When considering Θ together with the post-stimulus responses R in the same trial, we measured an increase in the information about stimuli (information gain, black line). The mean ± SEM across all experiments in basal condition are reported. The reported response representation is MUA.

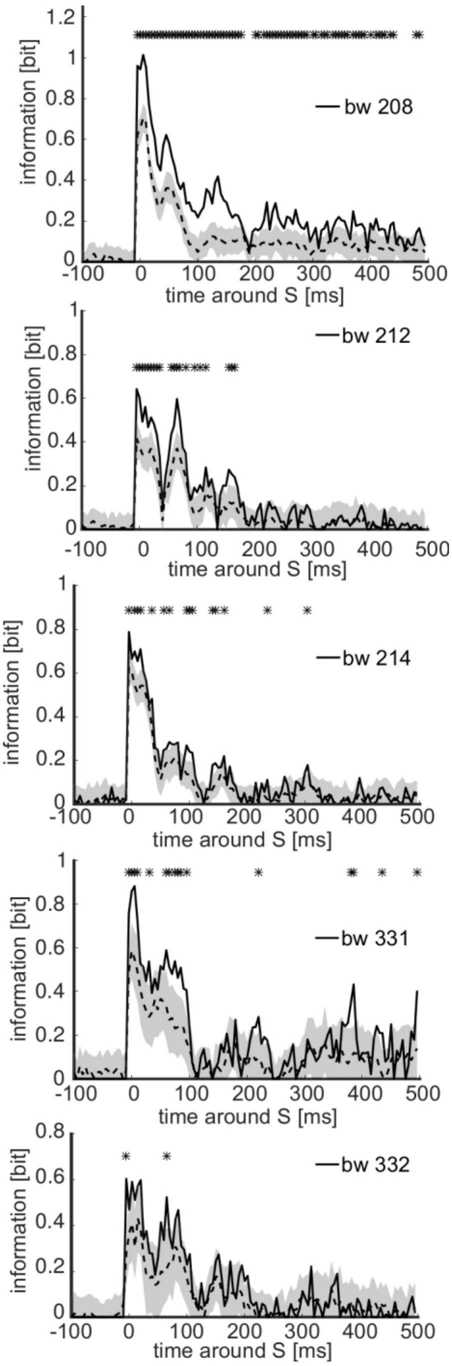

Supplementary Figure S2. Time course of information for different experiments in basal conditions. For each experiment, black solid line is the information $I(S;R,\Theta)$ about stimuli carried by the joint response features R=MUA and state variables Θ=TB, black dashed line is the information $I(S;R)$ between stimuli and responses and grey area delimits the 5th and 95th percentiles of the distribution $I(S;R,\Theta_{SH})$ obtained with N=200 random permutations of state variables Θ across trials. Stars indicate time points in which $I(S;R,\Theta)$ is significantly higher than

$I(S;R)$ (one-tailed permutation test, p<0.05, FDR corrected).

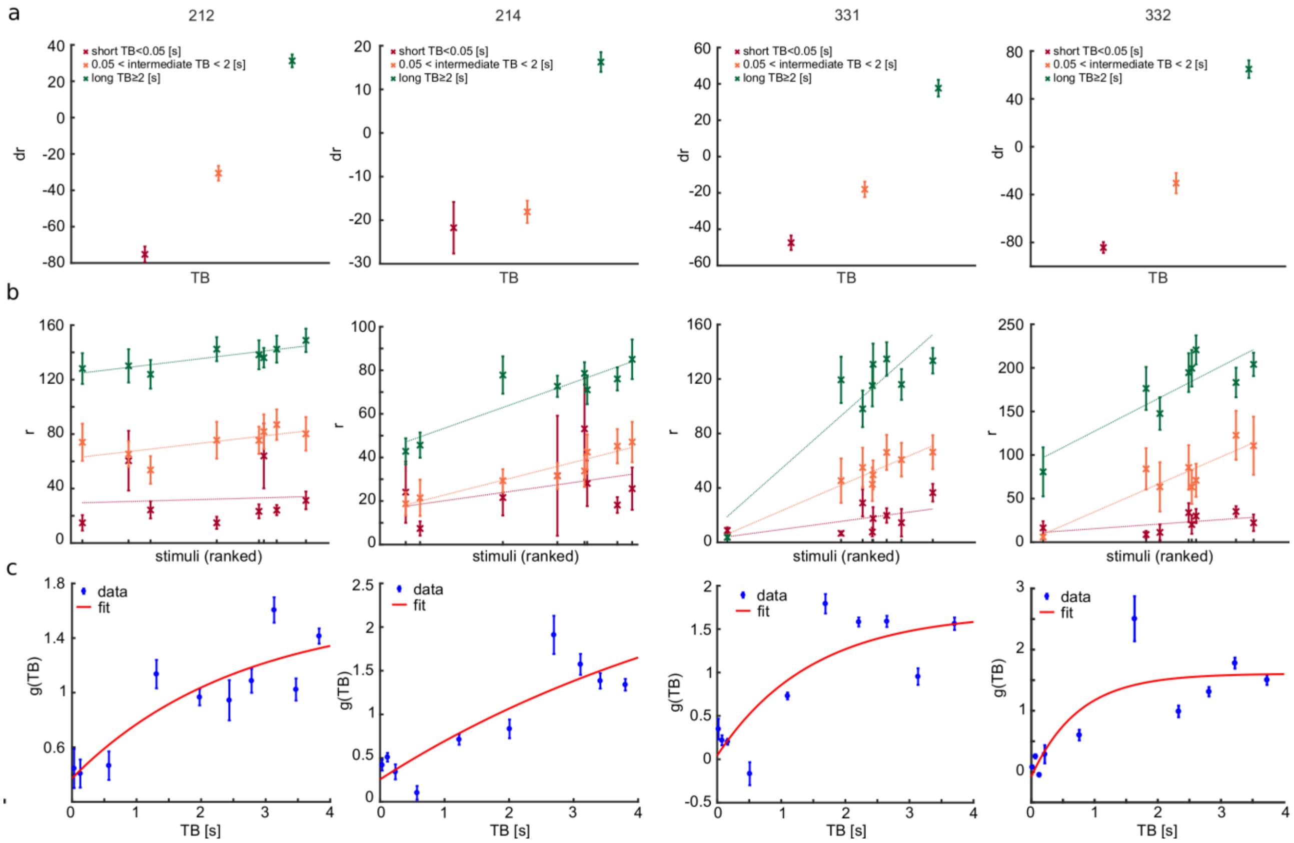

Supplementary Figure S3. Modeling single trial response as a function of state variable TB. Panels in different columns refer to results in different recording sessions (results for the example session 208 are reported in Fig. 4). **(a)** Mean ± SEM across trials of trial-to-trial variability dr of MUA as a function of TB, computed in [0 100] ms time interval after stimulus. State variable values are divided in three intervals: low TB if $\mathrm{TB} < 0.05\ s$ (red point), high TB (green point) if $\mathrm{TB} \geq 2\ s$, intermediate TB otherwise (yellow point). **(b)** Single trial response r as a function of the mean response at fixed stimulus $<r>_S$. Mean ± SEM across trials are reported for long, intermediate and short TB (lines represent best linear fits). **(c)** Mean ± SEM of $g(TB)$ computed in a 100 ms time window after the stimulus as a function state variable TB binned in 10 equi-populated intervals. Red line shows bi-exponential fit to the data points.

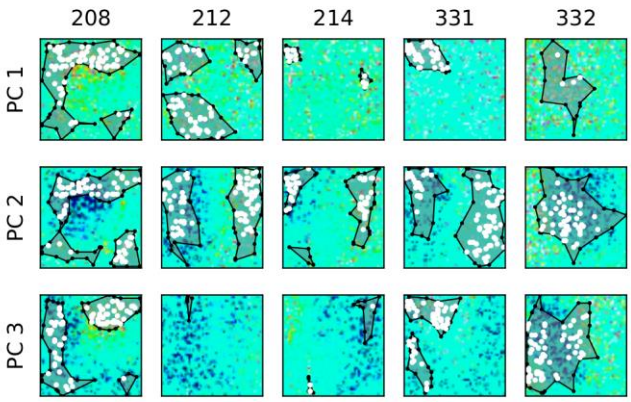

Supplementary Figure S4. The PCA reveals that cell cultures are characterized by specific regions in the network that respond more actively to electrical stimulation. The first three eigenvectors of the PCA decomposition are reported for five experiments (208,212,214,331,332) together with the electrodes selected with the $\sigma 3$ criteria (black and white circles). The shaded areas delimit the clusters of selected electrodes (black, on the border; white, within the area). The weights of the electrodes in the PCA maps are encoded by the colors (blue, negative; red positive). For a given experiment the shaded areas are quite comparable across the different PCs.

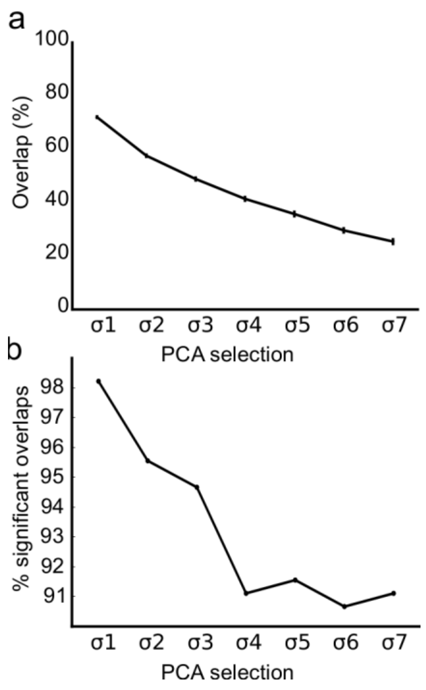

Supplementary Figure S5. The selected electrodes across different PCs are the same. (A) The overlaps (i.e. intersections) of the electrodes selected for different PCs decrease with increasing selection thresholds (from σ1 to σ7). (B) The overlap decreases with the selection criteria (from ~70%, for σ1, to ~20%, for σ7) but it is highly significant for most the computed overlaps (i.e. for more than the 90% of the computed overlaps, p<0.001, hyper-geometric test).

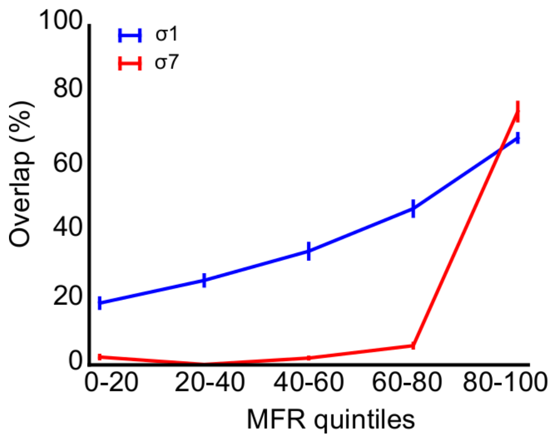

Supplementary Figure S6. The selected electrodes correspond to the most spontaneously firing ones. Plot of the mean ± sem over experiments of the overlap between the selected electrodes (i.e. thresholding the PC weights) in different quintiles of the distribution of mean firing

rates (MFR) within each experiment. The selected electrodes with σ7 (~300 electrodes) have approximately 80% overlap with the electrodes in the highest quintile of firing.

References

1. Banker, G. & Goslin, K. in *Cellular and molecular neuroscience series* (MIT Press, 1998). doi:36877411

2. Berdondini, L. *et al.* Active pixel sensor array for high spatio-temporal resolution electrophysiological recordings from single cell to large scale neuronal networks. *Lab Chip* **9,** 2644 (2009).

3. Bologna, L. L. *et al.* Low-frequency stimulation enhances burst activity in cortical cultures during development. *Neuroscience* **165,** 692–704 (2010).

4. Kermany, E. *et al.* Tradeoffs and constraints on neural representation in networks of cortical neurons. *J. Neurosci.* **30,** 9588–96 (2010).

5. Jimbo, Y., Kawana, A., Parodi, P. & Torre, V. The dynamics of a neuronal culture of dissociated cortical neurons of neonatal rats. *Biol. Cybern.* **83,** 1–20 (2000).

6. Gandolfo, M., Maccione, A., Tedesco, M., Martinoia, S. & Berdondini, L. Tracking burst patterns in hippocampal cultures with high-density CMOS-MEAs. *J. Neural Eng.* **7,** 56001 (2010).

7. Nieus, T., Di Marco, S., Maccione, A., Amin, H. & Berdondini, L. Investigating cell culture dynamics combining high density recordings with dimensional reduction techniques. *Proc. Annu. Int. Conf. IEEE Eng. Med. Biol. Soc. EMBS* 3759–3762 (2015). doi:10.1109/EMBC.2015.7319211

8. Panzeri, S., Senatore, R., Montemurro, M. A. & Petersen, R. S. Correcting for the sampling bias problem in spike train information measures. *J. Neurophysiol.* **98,** 1064–72 (2007).

9. Panzeri, S. & Treves, A. Analytical estimates of limited sampling biases in different

information measures. *Netw. Comput. Neural Syst.* **7,** 87–107 (1996).

10. Benjamini, Y. & Hochberg, Y. Controlling the false discovery rate: a practical and powerful approach to multiple testing. *J. R. Stat. Soc.* **57,** 289–300 (1995).

11. Quian Quiroga, R. & Panzeri, S. Extracting information from neuronal populations: information theory and decoding approaches. *Nat. Rev. Neurosci.* **10,** 173–85 (2009).

12. Schneidman, E., Bialek, W. & Berry, M. J. Synergy, Redundancy, and Independence in Population Codes. *J. Neurosci.* **23,** 11539–11553 (2003).

13. Kreuz, T., Mulansky, M. & Bozanic, N. SPIKY: a graphical user interface for monitoring spike train synchrony. *J. Neurophysiol.* **113,** 3432–3445 (2015).

14. Garofalo, M., Nieus, T., Massobrio, P. & Martinoia, S. Evaluation of the Performance of Information Theory-Based Methods and Cross-Correlation to Estimate the Functional Connectivity in Cortical Networks. *PLoS One* **4,** e6482 (2009).
